# Telomere-to-telomere genome assembly and a mutant library empower functional genomics and genetic improvement in *Cucurbita moschata*

**DOI:** 10.1016/j.xplc.2026.101836

**Published:** 2026-03-25

**Authors:** Juan Li, Zenghui Chen, Kui Li, Jingsheng Tan, Jing Sun, Xing Wang Deng, Younghoon Park, Hang He, Yun Deng, Xingping Zhang

**Affiliations:** 1 Department of Horticultural Bioscience, Pusan National University, Miryang 50463, Republic of Korea; 2 Peking University Institute of Advanced Agricultural Sciences, Shandong Laboratory of Advanced Agricultural Sciences at Weifang, Weifang, Shandong 261000, China; 3 Life and Industry Convergence Research Institute, Pusan National University, Miryang 50463, Republic of Korea

**Keywords:** *Cucurbita moschata*, T2T genome assembly, hybrid sterility, EMS mutant library, pollen mutagenesis, gene mining

## Abstract

Butternut squash (*Cucurbita moschata*) is an economically important crop; however, its genetic improvement has been hindered by the lack of high-resolution genomic resources and limited germplasm availability. In this study, we present a gap-free, telomere-to-telomere (T2T) genome assembly of *C. moschata* PKUMo, generated using high-accuracy Oxford Nanopore reads. The final assembly spans 314.34 Mb and is organized into 20 pseudomolecules, each represented by a single contig. Our analysis revealed that 40.58% of the genome consists of transposable elements, which have undergone significant expansion over the past 0.27 million years. Comparative genomic analysis with *Cucurbita maxima* (HZAU) identified substantial structural differences, including 27.20 Mb of inversions and 9.50 Mb of translocations, mainly affecting pericentromeric regions. We further investigated the evolution of centromeric regions in *C. moschata* and revealed distinct centromeric structures between PKUMo and HZAU. Notably, PKUMo centromeres exhibit increased transposon activity, particularly involving LTR retrotransposons. To facilitate functional genomics, we optimized an EMS-based pollen mutagenesis protocol, generating a mutant library comprising 60,000 M_1_ seeds and 800 M_2_ families, with 15.5% showing visible phenotypic variation. This library provides a valuable resource for dissecting agronomic traits and supports forward genetic approaches for identifying key genes in *C. moschata*. Using this T2T genome assembly, we successfully identified the causal genes *Cmos16G0077000* linked to a yellow-leaf phenotype and *Cmos14G0126400* associated with a miniature squash (mSq) phenotype. Overall, the PKUMo T2T genome assembly, together with the extensive mutant library, provides a robust foundation for exploring agronomic traits and accelerating genetic improvement in *Cucurbita* breeding programs.

## Teaser

This study reports a gap-free, telomere-to-telomere (T2T) genome assembly of *Cucurbita moschata* PKUMo, along with an analysis of structural variation relative to the closely related *Cucurbita maxima* genome. It also introduces the generation of a mutant library comprising 60,000 M_1_ seeds and 800 M_2_ families, of which 15.5% show visible phenotypic variation, using EMS-based pollen mutagenesis. Together, the T2T genome assembly and mutant library constitute valuable resources for gene discovery and molecular breeding in *Cucurbita* species.

## Introduction

tButternut squash, a popular variety of *Cucurbita moschata* within the genus *Cucurbita* (Kamiloglu et al., 2024), is widely cultivated for its nutritious and flavorful fruits, which are rich in vitamins, minerals, and dietary fiber (Dhiman et al., 2009; Dinu et al., 2016). However, the limited availability of genetic improvement resources for this type of squash has resulted in low average yields (FAO database, http://www.fao.org/). Wild squash and pumpkin varieties are typically characterized by bitterness (Kistler et al., 2015). Through domestication and selective cultivation, the flesh of pumpkins has gradually become the main edible part (Nee, 1990; Sanjur et al., 2002). However, this process has also led to the loss of traits associated with resistance to biotic and abiotic stresses to varying degrees (Barrera-Redondo et al., 2021; Jaccard et al., 2022). Therefore, it is essential to explore and utilize the genetic diversity of pumpkins to select and breed superior varieties with improved yield, quality, and stress resistance, thereby ensuring food security, meeting diverse consumer demands, and promoting sustainable agricultural development.

Three species—*C. maxima*, *C. moschata*, and *C. pepo*—are the primary cultivated *Cucurbita* species (Castellanos-Morales et al., 2018; Chomicki et al., 2020). *C. pepo*, commonly referred to as summer squash, is typically harvested for consumption while the fruit is still immature. Moreover, *C. pepo* is largely cross-incompatible with both *C. maxima* and *C. moschata*. Although interspecific hybrids of *C. maxima* and *C. moschata* are used as rootstocks or for fresh consumption, their high sterility severely limits breeding efforts aimed at integrating desirable traits from these two species.

Ethyl methanesulfonate (EMS), a widely used and efficient chemical mutagen, has been employed to induce genetic variation for gene discovery and to enrich genetic resources for breeding (Greene et al., 2003). The first EMS mutagenesis experiment in *C. pepo* was conducted in 2014 (Vicente-Dólera et al., 2014). In 2018, García et al. generated an EMS-mutagenized *C. pepo* population, with 10.82% of individuals exhibiting visible mutant phenotypes (García et al., 2018). Seeds are predominantly used in EMS mutagenesis experiments due to their ease of handling and manipulation. EMS mutant libraries have been constructed using seeds for various species, including rice, wheat, tomato, foxtail millet, zucchini, melon, and carrot (Galpaz et al., 2013; Vicente-Dólera et al., 2014; García et al., 2018; Sevanthi et al., 2018; Sun et al., 2019; Wu et al., 2020; Fonseca et al., 2022; Wang et al., 2023). However, to date, no EMS mutant library has been constructed or utilized for *C. moschata.* EMS mutant libraries generated from seeds are more resource-intensive and exhibit lower mutation rates than those produced via pollen mutagenesis (Deng et al., 2022). Pollen mutagenesis has been widely applied in maize research (Neuffer, 1994; Heuermann et al., 2019, Lu et al., 2018; Nie et al., 2021) and was later extended to watermelon and cucumber, where it has achieved considerable success (Deng et al., 2022; Tian et al., 2025).

A high-quality reference genome is crucial for studying gene function and enabling genetic improvement. Sun et al. assembled draft genomes of *C. maxima* and *C. moschata*, with sizes of 271.4 Mb and 269.9 Mb, respectively (Sun et al., 2017). The draft genome of *C. pepo* was first reported in 2018, with a size of 263 Mb (Montero-Pau et al., 2018). However, these genomes remained incomplete and contained numerous gaps that hindered in-depth genetic analyses. This limitation persisted until 2024, when Zeng et al. (2024) reported the first gap-free genome of *C. maxima.* The combination of a complete genome and a mutation library facilitates efficient identification of genes associated with specific phenotypes, thereby advancing functional genomics. To date, relatively few studies have been conducted in *C. moschata* (Abbas et al., 2020; Davoudi et al., 2022; Fu et al., 2024; Li et al., 2024). Therefore, it is essential to generate a telomere-to-telomere (T2T), gap-free genome of *C. moschata* and to establish a corresponding mutant library.

In this study, we generated a T2T, gap-free genome assembly for the butternut squash inbred line PKUMo (*C. moschata*) and constructed a mutant library from the same line using pollen EMS mutagenesis. Using this high-quality genome, genes associated with two mutants—a yellow-leaf mutant and a micro-dwarf mutant—were identified, highlighting the value of combining a T2T genome with an EMS mutant library for gene discovery and functional analysis. Ongoing investigations are examining additional mutations. This study provides valuable resources for the genetic improvement of *C*. *moschata*.

## Results

### Generation of a T2T, gap-free genome assembly of *C. moschata*

PKUMo is an elite inbred line of *C. moschata* specifically developed for butternut squash breeding. It was selected for this study for its desirable agronomic traits, including high yield, excellent taste, early flowering, strong fruit set, edible immature and mature fruits, and long shelf life.

**Figure 1:**
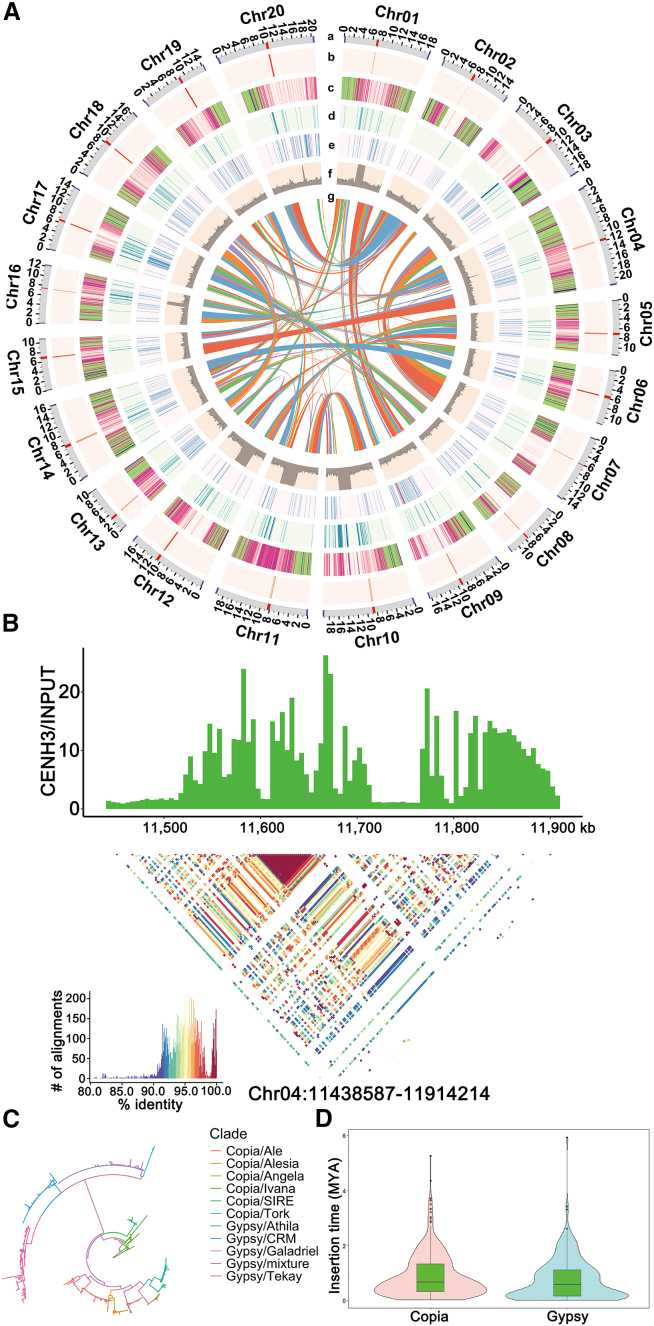
Overview of the telomere-to-telomere (T2T), gap-free reference genome assembly of butternut squash (PKUMo) **(A)** Circular diagram illustrating genomic features of the PKUMo assembly. Labels (a)–(g) indicate: (a) chromosomes with telomeres (purple) and centromeres (red); (b) distribution of CENH3 log_2_(ChIP/input) values along chromosomes; (c) density of high-confidence gene models; (d) density of full-length long terminal repeat (LTR)/Gypsy elements; (e) density of full-length LTR/Copia elements; (f) GC content; and (g) interchromosomal homologous relationships highlighted by central connecting lines. **(B)** Pairwise sequence identity heatmap of PKUMo centromeres. Non-overlapping 2 kb bins were aligned using Minimap2, retaining only the best local alignment for each bin pair. Sequence identity derived from the top-scoring alignments was visualized using StainedGlass, with the Chr04 centromere shown as an example. **(C)** Phylogenetic reconstruction of LTR retrotransposon (LTR-RT) subfamily diversification based on reverse transcriptase (RT) domains. **(D)** Distribution of insertion times for Copia and Gypsy retrotransposons.

Genome size was estimated to be 330.19 Mb based on 17-mer frequency analysis of Illumina sequencing data and 307.54 Mb via flow cytometry (Supplemental Figure 1), providing complementary validation across methods. For de novo assembly, Oxford Nanopore Technologies (ONT) ultra-long reads (>99.5% accuracy) and Hi-C reads were integrated using hifiasm, producing a highly contiguous assembly with a contig N50 of 16.50 Mb (Supplemental Tables 1 and 2). Chromosome-scale scaffolding was subsequently performed using yet another Hi-C Scaffolder (YaHS) with high-throughput chromosome conformation capture (Hi-C) data. To rigorously validate the hifiasm assembly and resolve residual gaps, independent assemblies were generated using Verkko, NextDenovo, and Flye, each leveraging combinations of high-fidelity (HiFi) and ONT data (Cheng et al., 2021; Freire et al., 2022; Hu et al., 2024; Supplemental Figure 2). Centromere positions were precisely mapped using CENH3 chromatin immunoprecipitation sequencing (ChIP-seq) data (Figure 1A and 1B), whereas telomeres were identified at chromosome termini based on the canonical plant telomeric repeat motif (AAACCCT). A total of 40 telomeres were detected, with telomeric repeat copy numbers ranging from 1,061 to 7,476 (Supplemental Table 3). The final assembly comprised 20 chromosome-length scaffolds spanning 314.34 Mb, with a contig N50 of 16.62 Mb (Figure 1A; Supplemental Tables 2 and 4), representing the first complete T2T, gap-free reference genome for *C. moschata*.

To comprehensively assess assembly quality, we performed a multi-dimensional evaluation. Mapping of raw HiFi and ONT reads to the assembly achieved exceptionally high alignment rates (99.78% and 99.88%, respectively) and near-complete genome coverage (99.77% and 99.91%; Supplemental Table 5; Supplemental Figure 6). Benchmarking Universal Single-Copy Orthologs (BUSCO) analysis against the embryophyta_odb10 database identified 98.5% (1,589 of 1,614) complete conserved genes, supporting high genomic completeness (Supplemental Table 6) (Simão et al., 2015). Merqury-based consensus quality value (QV) assessment yielded a score of 46.51, corresponding to an estimated base-level error rate of 0.00223% (Rhie et al., 2020). Hi-C contact maps showed no evidence of structural misjoins, even across previously challenging repetitive or gap-prone regions (Supplemental Figure 3). Furthermore, whole-genome collinearity analysis against the previously published *C. moschata* Rifu genome revealed extensive synteny (Supplemental Figure 4) (Sun et al., 2017). Notably, the PKUMo assembly (314.34 Mb) exceeds the Rifu genome (273.42 Mb) by 40.92 Mb in total length and by 76.48 Mb in chromosome-anchored sequence, highlighting substantial improvements in both contiguity and completeness (Supplemental Table 7).

Gene annotation predicted 28,594 protein-coding genes (PCGs), with a mean gene length of 3.22 kb and an average of 5.57 exons per gene (Supplemental Table 8). BUSCO assessment of the annotated gene set confirmed 96.0% completeness (Supplemental Table 9). Functional annotation assigned putative functions to 95.06% (27,357) of the PCGs through systematic homology searches across multiple curated databases (Supplemental Figure 5; Supplemental Table 10).

### TE annotations and evolutionary characteristics in the PKUMo genome

The PKUMo genome assembly contains 137.22 Mb of repetitive sequences, accounting for 43.65% of the total genome, which is substantially higher than the 38.35% reported for the Rifu genome. Of these repeats, 127.58 Mb (40.58% of the genome) were classified as transposable elements (TEs), including 157,995 long terminal repeat retrotransposons (LTR-RTs) and 7,936 DNA transposons (Supplemental Table 11). The Copia and Gypsy superfamilies were the most abundant, representing 9.89% and 7.50% of the genome, respectively. Additionally, 2,120 copies of 45S ribosomal DNA (rDNA) were identified, predominantly localized to Chr01, Chr10, Chr11, Chr12, and Chr16. In contrast, the single major 5S rDNA cluster was confined to Chr20 (Supplemental Table 12; Supplemental Figure 6).

To investigate the evolutionary dynamics of TEs, we identified full-length LTR-RTs and estimated their insertion times. A pronounced expansion of both Copia and Gypsy superfamilies occurred within the last 0.27 million years (Mya), as indicated by a sharp peak in the LTR divergence distribution (Figure 1C). We further classified LTR-RTs based on conserved reverse transcriptase (RT) domain sequences and constructed a phylogenetic tree (Figure 1D). The resulting tree revealed short, densely clustered branches within the Copia and Gypsy clades, including Ale, Angela, SIRE, CRM, Galadriel, Ogre, Reina, and Tekay, consistent with recent, lineage-specific amplification bursts (Supplemental Figure 7).

To assess the contribution of TEs to gene duplication, we classified 18,024 duplicated gene pairs into five structural categories: whole-genome duplication (WGD), tandem, proximal, transposed, and dispersed. WGD-derived pairs constituted the largest fraction (12,491 pairs; 69.30%), followed by dispersed (2,931; 16.26%), transposed (1,387; 7.70%), tandem (863; 4.79%), and proximal (352; 1.95%) duplicates. Analysis of synonymous substitution rate (Ks) distributions across these categories revealed significantly lower median Ks values for transposed, tandem, and proximal pairs compared with other types (Supplemental Figure 8), indicating that these duplicates arose more recently in the PKUMo lineage, likely through local, TE-facilitated or replication-based mechanisms rather than ancient polyploidization events.

### Genomic variation between PKUMo and *C. maxima* HZAU

**Figure 2:**
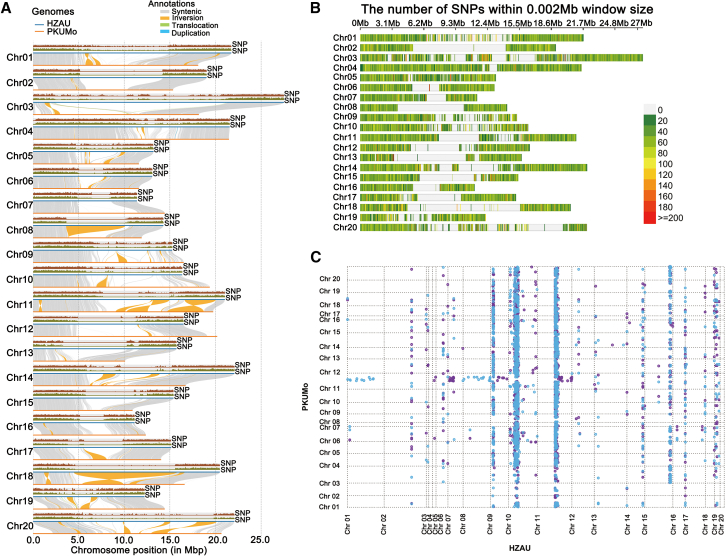
Differences between the *C. maxima* (HZAU) and *C. moschata* (PKUMo) genomes. **(A)** Large-scale genomic rearrangements and variant distributions across all 20 chromosomes (Chr01–Chr20) of HZAU (blue) and PKUMo (orange). Syntenic regions are shown in gray, with inversions and translocations highlighted in orange and green, respectively. Single-nucleotide polymorphisms (SNPs) and insertion–deletion variants (indels) are indicated by brown and green dots, respectively. **(B)** Genome-wide distribution of SNPs identified from the PKUMo–HZAU comparison, analyzed using a 2 kb sliding window. **(C)** Dot plot of centromeric regions between the PKUMo and HZAU genomes.

*C. maxima* is a genetically and agronomically important species within the genus *Cucurbita* (Zeng et al., 2024). We used SyRI to systematically characterize genomic divergence between the PKUMo genome and the HZAU reference assembly, including both large-scale structural variations (e.g., inversions, translocations, and duplications) and local variations (Figure 2A) (Goel et al., 2019). Synteny analysis revealed 152.18 Mb and 148.79 Mb of syntenic regions, 27.20 Mb and 35.58 Mb of inversion sequences, and 9.50 Mb and 9.41 Mb of translocated segments in PKUMo and HZAU, respectively (Supplemental Figure 9A). Within these aligned regions, we identified a total of 4,494,606 single-nucleotide polymorphisms (SNPs), corresponding to an average density of 12.98 SNPs per kilobase, calculated across non-overlapping 2-kb windows (Supplemental Figure 9B; Figures 2A and 2B).

Furthermore, we detected 23,968 and 20,263 unaligned regions in PKUMo and HZAU, spanning 110.99 Mb and 140.21 Mb, respectively (Supplemental Figure 9A). A total of 3,739 genes were identified within the PKUMo unaligned regions. Gene Ontology (GO) enrichment analysis revealed significant overrepresentation of terms related to terpene metabolism (including mono-, sesqui-, and general terpene biosynthetic and metabolic processes), secondary metabolite biosynthesis, reproductive processes (e.g., pollination and pollen–pistil interaction), and transposable element activity (retrotransposition) (Supplemental Figure 10).

Comparative structural analysis identified 171 inversion regions in the PKUMo genome, distributed across all 20 chromosomes. Of these, 21 were inter-arm inversions, located on Chr01 (*n* = 2), Chr03 (*n* = 1), Chr04 (*n* = 2), Chr05 (*n* = 1), Chr06 (*n* = 1), Chr09 (*n* = 1), Chr11 (*n* = 3), Chr13 (*n* = 1), Chr14 (*n* = 3), Chr17 (*n* = 2), Chr18 (*n* = 2), and Chr20 (*n* = 2). Inversion sizes ranged from 103 bp to 4,523,369 bp (mean = 159,056.30 bp), with seven regions exceeding 1 Mb: Chr10 (1,081,590 bp), Chr11 (1,005,794 bp; 2,274,977 bp; and 3,983,875 bp), Chr17 (1,501,254 bp), Chr18 (4,523,369 bp), and Chr20 (1,767,479 bp). A total of 1,645 genes were annotated within these inversion intervals. GO enrichment analysis revealed significant enrichment of molecular functions related to protease binding, various enzyme activities (e.g., glucosidase, beta-glucosidase, and pectinesterase activities), hormone activity, and multiple signaling- and peptidase-related regulatory activities (including signaling receptor regulator activity, signaling receptor activator activity, endopeptidase inhibitor activity, and peptidase inhibitor activity) (Supplemental Figure 11).

### Characterization and evolution of centromeric regions

The centromere is a critical chromosomal domain required for kinetochore assembly and spindle microtubule attachment, thereby ensuring faithful chromosome segregation during mitosis and meiosis (Comai et al., 2017). Centromere identity is epigenetically specified by the centromere-specific histone H3 variant CENH3, which serves as a foundational determinant of centromere function (Liu et al., 2023). To precisely define core centromeric boundaries across all 20 chromosomes, we performed CENH3-directed ChIP-seq (Supplemental Table 13). Distinct chromosome-wide ChIP-seq peaks were detected on each chromosome, supporting the completeness and structural integrity of centromere assembly (Figure 1A).

Collinearity analysis revealed low sequence conservation between the centromeric regions of PKUMo and HZAU (Figure 2C). To further characterize this divergence, we performed tandem repeat annotation using pyTanFinder. The results showed that PKUMo centromeres were predominantly composed of three major monomeric repeats: CEN90 (90 bp), CEN168, and CEN197 (Supplemental Table 14). In contrast, HZAU centromeres were enriched in six distinct tandem repeat families: CEN169, CEN253, CEN315, CEN324, CEN327, and CEN654 (Zeng et al., 2024). This pronounced compositional divergence indicates fundamentally distinct evolutionary trajectories of centromeric repeat organization between the two genomes.

LTR-RTs are known to preferentially accumulate in plant centromeres (Naish and Henderson, 2024). In PKUMo, we identified 1,192 intact LTR-RTs, 124 of which were located within centromeric regions (Supplemental Table 15). Insertion time estimation showed that centromeric LTR-RTs had a median age of ∼0.19 Mya, significantly younger than non-centromeric LTR-RTs (∼0.30 Mya), suggesting recent, centromere-biased retrotranspositional activity. Compared with HZAU, centromeric LTR-RTs in PKUMo exhibited younger insertion ages, implying lineage-specific bursts of retrotransposition within centromeric regions.

### Phenotypic and genetic evaluation of the PKUMo EMS mutant library

**Figure 3:**
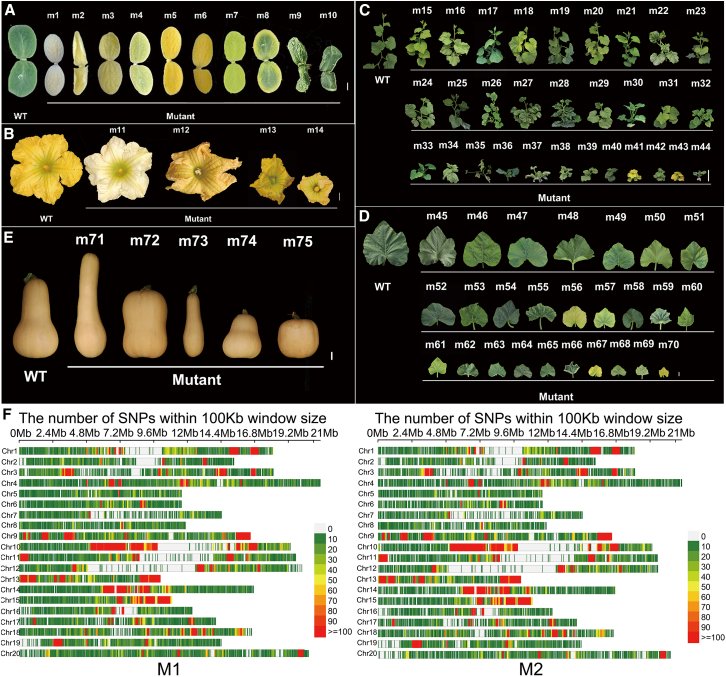
Phenotypic and genotypic variation in the PKUMo EMS mutant library. **(A)** Cotyledon mutants. **(B)** Male flower mutants. **(C)** Plant architecture mutants. **(D)** Leaf mutants. **(E)** Fruit shape mutants. **(F)** Distribution and density of mutations identified in 38 M_1_ and 30 M_2_ mutants. A 100 kb window was used, with color intensity indicating mutation frequency. Scale bars: **(A)**, **(B)**, **(D)**, and **(E)**, 1 cm; **(C)**, 10 cm.

Among the 800 M_2_ families derived from EMS-mutagenized PKUMo, 124 (15.5%) exhibited significant phenotypic alterations. These mutant phenotypes manifested across all developmental stages and were classified into six distinct categories based on primary morphological features (Figure 3A–3E; Supplemental Table 16). Variations in plant architecture, leaf morphology, and leaf color were the most prevalent, collectively observed in 6.5% of all M_2_ families. A total of 113 mutants displayed alterations in one or more of these three traits. Notably, most variants have not been previously reported in *C. moschata*.

Most chlorotic mutants exhibited seedling lethality; however, mutant line m15 showed stable, uniform yellow pigmentation from the cotyledon stage onward, flowered normally, set viable fruit, and faithfully transmitted the trait across generations. In addition, a strong dwarf mutant, m27, was identified among the M_2_ families. This mutant exhibited severely compact growth, predominantly produced male flowers, and formed only rudimentary, non-expanding female flowers incapable of fruit set. Crosses with the wild-type parent yielded F_1_ progeny indistinguishable from the wild type, consistent with recessive monogenic inheritance. Another mutant lacked viable pollen in male flowers and failed to produce seeds even after manual pollination with wild-type pollen. Mutants with altered fruit morphology were also recovered, including lines exhibiting significantly elongated or oblate fruits relative to the wild type. Some mutants showed changes in more than one trait. For example, mutant 23SQ070 (Supplemental Figure 12) segregated independently for two unlinked traits—plant morphology and color. Both traits followed classic Mendelian recessive segregation ratios (3:1) in the M_2_ generation, indicating discrete single-locus mutations. Collectively, these observations suggest that multiple independent mutational events may be present within some M_2_ families, warranting further investigation.

Whole-genome sequencing of M_1_ and M_2_ individuals revealed mean SNP densities of 5190.05 and 6306.83 per sample, corresponding to average mutation rates of 15.96 and 19.46 SNPs per Mb, respectively (Figure 3F; Supplemental Table 18). A→G substitutions were most frequent in M_1_ plants, whereas C→T transitions were enriched in M_2_ plants (Supplemental Table 17).

With a sufficiently large mutant population, near-saturation of gene function can be achieved. Among 30 sequenced M_2_ mutants, we identified 5,755 high-confidence coding-region mutations. On average, each mutant carried 191.8 mutations in coding regions. Based on the 28,594 genes annotated in the PKUMo genome, a library comprising 1,200 mutants is predicted to achieve a ≥ 99% probability of introducing at least one loss-of-function allele in every gene. We have generated over 60,000 M_1_ seeds, providing sufficient mutational load to achieve near-complete coverage of agronomically important genes in the genome.

### Gene discovery and molecular marker development for the yellow-leaf mutant

**Figure 4:**
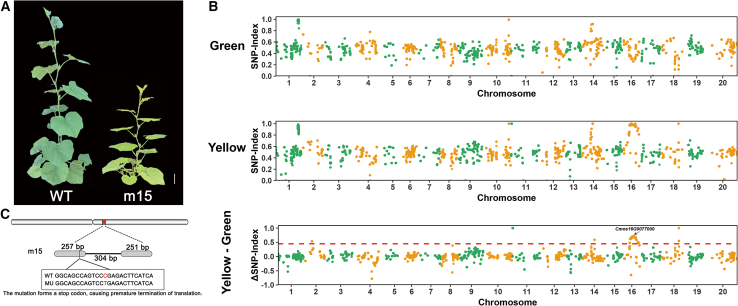
Genetic mapping of the yellow-leaf mutant m15. **(A)** Phenotypes of the wild type and m15 mutant. **(B)** SNP-index plots from QTL-seq analysis, showing the green bulk (normal phenotype), yellow bulk (etiolated phenotype), and Δ(SNP-index) (yellow minus green). The *x*-axis represents physical positions (Mb) across the 20 PKUMo chromosomes (chromosomes lacking valid data were omitted), and the *y*-axis indicates SNP-index values. The SNP-index was calculated using a 200 kb window with a 10 kb sliding step. Δ(SNP-index) was plotted with a 99% confidence interval (*p* < 0.01) under the null hypothesis of no QTL. A total of 16 QTLs were identified on chromosomes 02, 11, 14, 16, and 18 (220–610 kb intervals) based on the following criteria: SNP-index close to 0 in the green bulk, SNP-index close to 1 in the yellow bulk, and Δ(SNP-index) exceeding the confidence threshold of 0.45 (*p* < 0.01). The experimentally validated candidate region was mapped to the *Cmos16G0077000* gene on Chr16. **(C)** Gene structure comparison between the wild type and m15 mutant. Scale bar, 10 cm.

Leaf color is a key agronomic and physiological trait, serving as both a visual indicator of photosynthetic capacity and a practical marker for varietal purity assessment. As the primary photosynthetic organs, leaves are essential for plant growth and development. Although most chlorotic mutants are seedling lethal, mutant m15 represents a viable yellow-leaf variant that completes its life cycle, including normal flowering, pollination, and fruit set (Figure 4A). Self-pollination of m15 produced uniformly yellow-leaved M_3_ progeny, confirming stable homozygosity. Genetic analysis further showed that all F_1_ hybrids from crosses between m15 and the wild type exhibited green leaves, indicating that the yellow-leaf phenotype is recessive. In the F_2_ generation (*n* = 216), segregation yielded 156 green-leaved and 60 yellow-leaved plants—consistent with the expected 3:1 Mendelian ratio (χ^2^ = 0.89 < χ^2^_0_._05_,_1_ = 3.84), thereby confirming monogenic recessive control of leaf etiolation, with green leaf color dominant over yellow.

Integrated bulked segregant analysis sequencing (BSA-seq) and genetic mapping identified *Cmos16G0077000* as a candidate gene controlling etiolation in PKUMo (Figure 4B). This locus encodes LHCP TRANSLOCATION DEFECT (LTD), a chloroplast-localized protein that interacts with both the signal recognition particle (SRP) pathway and the inner chloroplast envelope (Ouyang et al., 2011). Functional enrichment analysis revealed that downregulated genes were significantly associated with leaf senescence and karrikin response (Supplemental Figure 13), suggesting a potential role for these genes in the regulation of leaf development.

Whole-genome resequencing aligned to the PKUMo assembly identified a single G-to-A transition at nucleotide position 203 within the coding sequence of *Cmos16G0077000*. This mutation introduces a premature stop codon, resulting in a truncated and likely nonfunctional LTD protein (Figure 4C). Based on this SNP, we developed a Kompetitive Allele Specific PCR(KASP) marker for high-throughput genotyping. In validation assays, the wild-type allele produced a blue fluorescence signal, whereas the homozygous mutant (m15) allele produced a distinct red signal (Supplemental Figure 14). To further validate this marker, we genotyped individual F_2_ plants at the target site (Supplemental Figure 15). The KASP marker co-segregated with leaf color: in the F_2_ population, yellow mutants consistently produced red signals, whereas green plants produced green or blue signals at an approximate 2:1 ratio (green:blue). The leaf color phenotypes were highly consistent with the genotyping results, confirming that this KASP marker reliably and efficiently distinguishes genotypes at the target site in progeny.

### Gene discovery and molecular marker development for the micro-plant mutant

**Figure 5:**
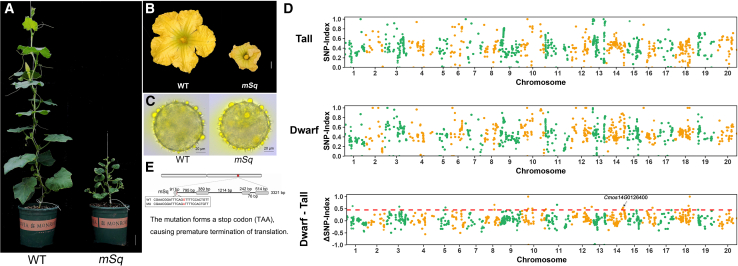
Genetic mapping of the dwarf mutant *mSq*. **(A)** Phenotypes of the wild type and mSq mutant. **(B)** Male flower phenotypes of the wild type and mSq mutant. **(C)** Pollen morphology of the wild type and mSq mutant. **(D)** SNP-index plots from QTL-seq analysis, showing the tall bulk (normal phenotype), dwarf bulk (dwarf phenotype), and Δ(SNP-index) (dwarf minus tall). The *x*-axis represents physical positions (Mb) across the 20 PKUMo chromosomes (chromosomes lacking valid data were omitted), and the *y*-axis indicates SNP-index values. The SNP-index was calculated using a 200 kb window with a 10 kb sliding step. Δ(SNP-index) was plotted with a 99% confidence interval (*p* < 0.01) under the null hypothesis of no QTL. A total of 17 QTLs were identified on chromosomes 01, 03, 05, 08, 10, 11, 12, 14, 16, 18, and 19 (200–390 kb intervals) based on the following criteria: SNP-index close to 0 in the tall bulk, SNP-index close to 1 in the dwarf bulk, and Δ(SNP-index) exceeding the confidence threshold of 0.45 (*p* < 0.01). The experimentally validated candidate region was mapped to the *Cmos14G0126400* gene on Chr14. **(E)** Gene structure comparison between the wild type and *mSq* mutant. Scale bar, 10 cm.

Plant architecture is a key agronomic trait in butternut squash breeding. Dwarf plants can effectively reduce row spacing, thereby increasing planting density and potentially improving yield per unit area. In the M_2_ population, we isolated a dwarf mutant (m27, hereafter designated *mSq*) that exhibited a pronounced male-flower phenotype. The mutant rarely produced female flowers, and those that did develop were morphologically abnormal or failed to develop properly, preventing the production of self-pollinated seeds (Figure 5A). Notably, although male flowers of *mSq* were smaller than those of the wild type, pollen viability and germination capacity were unaffected (Figures 5B, 5C). When male flowers of the *mSq* mutant were crossed with the wild type, the F_1_ progeny displayed the wild-type phenotype. Dwarf plants were observed in the F_2_ populations, and the segregation ratio of normal to short plants in three independent F_2_populations conformed to the expected 3:1 Mendelian ratio (Supplemental Table 18), providing strong genetic evidence that the *mSq* phenotype is controlled by a single recessive nuclear locus.

To identify the causal gene underlying the dwarf and female-sterile phenotype, we performed BSA using DNA pools derived from 20 extreme dwarf plants and 20 wild-type-like individuals from the F_2_ population. This analysis mapped the candidate region to *Cmos14G0126400* (Figure 5D), which is annotated to encode β-1,4-xylosyltransferase IRX10. Because xylosyltransferases are essential for cell wall biosynthesis, loss of IRX10 function is expected to impair cell wall formation, potentially resulting in reduced size or developmental defects in stems, leaves, and flowers (Hörnblad et al., 2013). Therefore, IRX10 represents a strong candidate underlying the mutant’s dwarfism and female sterility. Whole-genome resequencing identified a G-to-A mutation in the dwarf mutant that causes premature translation termination in the deduced protein (Figure 5E). A molecular marker derived from this mutation further validated that the observed phenotypes co-segregated with this locus in the F_2 population_ (Supplemental Figure 16).

### PumpkinDB: An integrated database for *C. moschata*

We constructed PumpkinDB (http://omicsplant.cn/Cucurbitamoschata/), a comprehensive resource database for *C. moschata*, by integrating all genomic and transcriptomic datasets generated in this study. The database comprises three core functional modules: Download, Mutant, and Search. The Download module provides free access to key multi-omics resources, including the T2T *C. moschata* reference genome (PKUMo) and its corresponding gene annotations, genome-wide variant loci identified from EMS-induced mutant lines through whole-genome resequencing, and raw sequencing datasets (PacBio HiFi, ONT long reads, and RNA-seq). The Search module enables visualization and browsing of genome annotations and supports gene queries by gene ID. The Mutant module provides curated phenotypic records and images from the EMS mutant library, with all mutants systematically classified into diverse trait categories, and allows users to retrieve detailed information on specific mutants by name. In summary, PumpkinDB features a unified and user-friendly interface and serves as an integrated multi-omics resource platform that facilitates gene mining, functional genomics research, and molecular breeding applications in *C*. *moschata*.

## Discussion

This study constructed a T2T gap-free reference genome for the inbred line PKUMo of *C. moschata* by integrating multi-platform sequencing data and employing multiple assembly strategies. The resulting assembly achieves high quality across multiple metrics compared with existing *C. moschata genomes*. Our analysis indicates that LTR-RTs of the *Copia* and *Gypsy* superfamilies have undergone substantial expansion over the past 0.27 million years (e.g., a recent burst of the SIRE clade) (Supplemental Figure 7), and that LTR-RTs in centromeric regions (with an average insertion time of 0.19 Mya) are younger than those in non-centromeric regions (0.30 Mya). These results suggest that recent activity of LTR-RTs may drive the evolution of genome size in butternut squash through transposon bursts and may also contribute to the epigenetic regulation of centromeres. This provides direct evidence from the genus *Cucurbita* supporting the LTR-RT-driven hypothesis of plant centromere evolution (Volff, 2006; Usha et al., 2022; Heuberger et al., 2024).

Hybrid sterility between *C. moschata* and *C. maxima* represents a critical bottleneck restricting the utilization of interspecific genetic resources in *Cucurbita*. Accurate pairing of homologous chromosomes during meiosis is essential for gamete formation (Chriss et al., 2024). Numerous inversions and translocations are present between PKUMo and HZAU, primarily concentrated in pericentromeric regions. The centromeres of PKUMo are predominantly composed of repetitive units such as CEN90, CEN168, and CEN197 (Supplemental Table 14), whereas those of HZAU mainly consist of CEN169, CEN253, CEN315, CEN324, CEN327, and CEN654, with little sequence collinearity between them. These structural differences may lead to the formation of inversion loops in homologous chromosomes of inverted regions during meiosis in hybrid offspring, potentially causing chromosome breakage or bridging. Furthermore, mismatched centromeric repetitive sequences may impair CENH3-mediated centromere recognition, resulting in abnormal spindle microtubule attachment (Comai et al., 2017). Ultimately, these defects disrupt chromosome segregation in gametes, thereby reducing the proportion of fertile gametes. This is consistent with the observed high pollen abortion rates in interspecific F_1_ hybrids in the field. In addition to structural variation, SNPs within collinear regions and species-specific genes in unaligned regions may further exacerbate interspecific reproductive isolation. Within the 110.99 Mb unaligned region of PKUMo, 3,739 genes are enriched in functional categories related to secondary metabolism, reproductive processes, and transposable element activity, which may further impair fertilization and exacerbate hybrid sterility.

Abnormal activation of transposable elements in plants can disrupt epigenetic regulation and, in turn, affect gene expression. In this study, the insertions of LTR-RTs in PKUMo, particularly in centromeric regions, were relatively recent, whereas most LTR-RTs in HZAU represent ancient insertion events. This difference may lead to the activation of young LTR-RTs from PKUMo in the genetic background of HZAU. These activated LTR-RTs can insert into gene bodies or regulatory regions, resulting in gene silencing.

Unlike most chlorotic mutants that are seedling lethal, m15 exhibits consistent yellowing from the seedling to the fruiting stage and can set fruit normally (Figure 4A). By integrating BSA-seq with the T2T genome, the candidate gene *Cmos16G0077000*, which encodes LTD, was mapped (Figure 4B). LTD is a key component of the chloroplast SRP pathway and is responsible for transporting light-harvesting chlorophyll-binding proteins (LHCPs) of photosystem II (Ouyang et al., 2011). Mutation of LTD impairs the transport of LHCPs into chloroplasts, disrupting chlorophyll biosynthesis and resulting in a yellow-leaf phenotype (Zhang et al., 2022b, 2025).

The candidate gene *Cmos14G0126400* underlying *mSq* encodes the IRX10 protein (Figure 5D). IRX10 is involved in xylan biosynthesis in plant cell walls; loss of its function leads to abnormal cell wall structure, thereby affecting stem elongation and floral organ development (Hörnblad et al., 2013). The identification of this mutant not only reveals a coordinated regulatory mechanism linking plant architecture and flower development in pumpkin but also provides a novel genetic target for breeding dwarf varieties, which are well suited for mechanized cultivation and high-density planting (Dou et al., 2023).

## Methods

### Plant materials

A high-quality inbred line of butternut squash, PKUMo, was selected for genome assembly and mutant library construction. Plants were cultivated at the experimental station of the Peking University Institute of Advanced Agricultural Sciences in Weifang, Shandong Province (36°50′N, 119°44′E) under standard agricultural practices.

### Library construction and sequencing

Genomic DNA (gDNA) was isolated from true leaves of 2-week-old seedlings using the cetyltrimethylammonium bromide (CTAB) method. For PacBio HiFi library construction, samples containing more than 5 μg of fragmented DNA were subjected to size selection using the BluePippin system, followed by concentration with AMPure PB beads. HiFi SMRTbell libraries were constructed using the SMRTbell Express Template Preparation Kit 2.0 (Pacific Biosciences, CA, USA) and sequenced on the PacBio RS II circular consensus sequencing (CCS) platform. For ONT ultra-long sequencing, libraries were prepared following the standard protocol using the SQK-LSK114 ligation kit. The purified libraries were loaded onto pre-primed R10.4.1 Spot-On flow cells and sequenced on the PromethION platform.

For Hi-C library construction, cells were crosslinked with formaldehyde, and DNA was digested with the 4-cutter restriction enzyme *Dpn*II. The ends were then filled in and biotin-labeled, followed by ligation of blunt-end fragments and DNA purification. The purified DNA was randomly sheared into 300–500 bp fragments. The libraries were preliminarily quantified using Qubit 2.0, insert sizes were assessed using an Agilent 2100 system, and effective concentrations were quantified by qPCR. After quality control, sequencing was performed on the Illumina NovaSeq 6000 platform.

RNA was extracted from various tissues, including young stems, mature leaves, young leaves, seeds, pollen, and radicles. RNA integrity, quality, and concentration were evaluated using a NanoDrop 2000 spectrophotometer (Thermo Fisher Scientific) and an Agilent 2100 Bioanalyzer (Agilent Technologies). Illumina RNA-seq libraries were prepared using the NEBNext Ultra RNA Library Prep Kit following the manufacturer’s protocols and sequenced on an Illumina NovaSeq 6000 platform. For single-molecule real-time isoform sequencing (Iso-Seq) sequencing, full-length cDNA was synthesized using a SMARTer PCR cDNA Synthesis Kit (Takara Biotechnology, Dalian, China). Equal amounts of cDNA from different tissues were pooled to construct a mixed sample. SMRTbell libraries were prepared using the SMRTbell Express Template Prep Kit 2.0 (Pacific Biosciences) and sequenced on the Sequel II system.

For mutation library analysis, DNA was extracted from leaves using the CTAB method. DNA quality was assessed by NanoDrop spectrophotometry and 1% agarose gel electrophoresis, and concentration was quantified using a Qubit DNA Assay Kit and a Qubit 3.0 fluorometer (Invitrogen, USA). Libraries were prepared using the NEBNext Ultra DNA Library Prep Kit for Illumina (New England Biolabs, MA, USA) and sequenced on an Illumina NovaSeq 6000 platform.

### Gap-free genome assembly with PacBio HiFi and ONT ultra-long reads

Before de novo genome assembly, the genome size of PKUMo was estimated using k-mer frequency distribution analysis. Jellyfish (v2.1.1) was used to generate 17-mer frequency counts (Marçais and Kingsford, 2011), and findGSE (v0.1.0; Sun et al., 2018) was employed to infer genome size and repeat content based on these k-mers.

For flow cytometry-based genome size estimation, 1 g of mature leaves was weighed and placed in a sterile Petri dish containing 1 ml of ice-cold Otto I buffer (0.1 M citric acid, 0.5% Tween 20). The suspension was filtered through a Partec CellTrics 40 μm filter to remove tissue debris and incubated for at least 10 min at room temperature. The isolated nuclei in the filtered suspension were stained with 1 ml of Otto II buffer (0.4 M Na_2_HPO_4_·12 H_2_O) containing the AT-specific fluorochrome 4′,6-diamidino-2-phenylindole (DAPI; 4 μg ml^−1^) and β-mercaptoethanol (2 μg ml^−1^) (Paule et al., 2018). Using the known genome size of watermelon G42 as a reference, the genome size of PKUMo was calculated based on the ratio of relative fluorescence intensity between PKUMo and G42.

Five genome assemblers with distinct algorithms were employed for PKUMo genome assembly: (1) Verkko (v1.4.1; Rautiainen et al., 2023) using PacBio HiFi, ONT ultra-long reads, and Hi-C data (parameters: --base-k 3001 --window 2500); (2) hifiasm (v0.19; Cheng et al., 2021), integrating PacBio HiFi, ONT ultra-long reads, and Hi-C data (parameter: --max-kocc 4000); (3) Hi-Canu (v2.2; Koren et al., 2017) using PacBio HiFi reads (parameters: genomeSize = 330 m, minReadLength = 4000, minOverlapLength = 3000, corMaxEvidenceErate = 0.15); (4) Flye (v2.9.1; Freire et al., 2022); and (5) NextDenovo (v2.5.2; Hu et al., 2024), both using ONT ultra-long reads with default parameters. Redundant sequences were removed using Purge_haplotigs (v1.0.4; Roach et al., 2018). Contigs generated by hifiasm were anchored to chromosomes using YaHS (v1.2.2; Zhou et al., 2023) with Hi-C data, and assembly accuracy was validated through collinearity analysis with *Cucurbita moschata* (Rifu). Only one gap remained on Chr10 after validation. Assemblies generated by Verkko, Hi-Canu, Flye, and NextDenovo were aligned to Chr10; contigs spanning the gap were manually inspected, and the gap was filled using the GapFiller module of quarTeT (v1.1.5) (Lin et al., 2023). Missing telomeres were recovered using Teloclip (https://github.com/Adamtaranto/teloclip) based on these assemblies, and chromosome-end telomeres were identified using the TeloExplorer module of quarT2T.

### Assessment of assembly quality

To comprehensively assess genome assembly quality, including completeness and sequencing uniformity, PacBio HiFi and ONT ultra-long reads were aligned to the assembled genome using Minimap2 (v2.24) (Lu et al., 2018). Two key metrics—mapping rate and genome coverage—were quantified using the flagstat and coverage subcommands of SAMtools (v1.19), respectively (Danecek et al., 2021). For downstream read-depth analysis, primary alignments (FLAG = 0 or 16 in SAM files) were retained. Per-base read depth was calculated using BEDTools (v2.29.2), and average read depth was computed across 1-kb genomic bins (Quinlan and Hall, 2010). Genome assembly quality was further evaluated by estimating the QV score using HiFi reads with Merqury (Rhie et al., 2020). Gene completeness was assessed using BUSCO v5.4.5 with the embryophyta_odb10 dataset. Hi-C data were aligned to the assembly using Hi-C-Pro v2.7.0 (Servant et al., 2015), and the resulting contact matrix was visualized using custom Python scripts.

### Collinearity analysis

For collinearity analysis, the *C. moschata* PKUMo and Rifu genomes were aligned using NUCmer with the parameters “--mum --mincluster = 200 --minmatch = 100” (Marçais et al., 2018). Alignment results were subsequently filtered using the parameters “-i 95 -L 100-1” to retain 1-to-1 alignments. Collinearity was visualized as a dot plot using a custom R script.

### rDNA identification

Barrnap (v0.9) (https://github.com/tseemann/barrnap) was used to annotate ribosomal RNA genes with the parameter “--kingdom euk.” Additionally, rDNA sequences were identified in HiFi reads, and the copy number of rDNAs in PKUMo was estimated to be ∼2,192 (181,983/83) based on rDNA-containing HiFi reads.

### Annotation of repeats

Repeat elements in the PKUMo genome were annotated using a combination of *de novo* prediction and homology-based approaches. Known repeat sequences were first retrieved from Dfam (v3.8) using “Cucurbitaceae” as the keyword. A de novo repeat library was then constructed using RepeatModeler (Flynn et al., 2020). These datasets were merged, and redundancies were removed using CD-HIT to generate a non-redundant repeat library (Fu et al., 2012). Finally, RepeatMasker (http://www.repeatmasker.org/) was used to annotate genomic repeat regions based on this non-redundant library.

### Identification of centromeric regions

ChIP assays were performed using anti-CENH3 antibodies (PHYTOAB, PHY6615A) as previously described (Sun et al., 2024). The ChIP-enriched and input DNA samples were used to construct sequencing libraries according to the protocols of the VAHTS Universal DNA Library Prep Kit for Illumina V4 (Vazyme, ND610) and the VAHTS Multiplex Oligos Set 4 for Illumina (Vazyme, N321). Libraries were sequenced on the Illumina NovaSeq platform (150 bp paired-end reads).

Quality control of ChIP-seq raw reads was performed using fastp (v0.23.4) to remove low-quality bases and adapter sequences (Chen et al., 2018). Filtered reads were aligned to the PKUMo genome assembly using Bowtie2 (v2.5.4) with default parameters (Langmead et al., 2019). The resulting BAM files were processed using SAMtools (v1.19) to remove unmapped reads and PCR duplicates. Peak calling was performed using MACS3 (v3.0.3) (Zhang et al., 2008), with active centromeres defined as genomic regions exhibiting > 2-fold enrichment (FC > 2) relative to input controls. For precise centromere boundary annotation, ChIP-seq signals were normalized against input using bamCompare (v3.5.6) with the following parameters: --ignoreDuplicates -- scaleFactorsMethod None --normalizeUsing RPKM. Regions with an enrichment ratio > 2.5 were designated as candidate centromeres and merged within 200-kb windows using BEDTools (v2.30). Finally, centromere positions were manually verified and refined using the Integrative Genomics Viewer (IGV) (Robinson et al., 2011), and centromeric repeats were visualized using StainedGlass (v0.6) (Vollger et al., 2022). Tandem repeats within centromeric regions were annotated using pyTandem with default parameters (Kirov et al., 2018).

### Gene prediction and functional annotation

The protein-coding gene structures in the PKUMo genome were predicted using a combined approach integrating *ab initio*, transcriptome-based, and homology-based methods.

For transcriptome-based prediction, both RNA-seq and Iso-Seq data were utilized. RNA-seq reads were mapped to the PKUMo genome using STAR (v2.7.11a) (Dobin et al., 2013). The resulting BAM files were merged into a single alignment file using SAMtools (v1.19) and assembled into transcripts using StringTie (v2.2.1; parameters: -m 150 -f 0.1 -t) (Danecek et al., 2021; Pertea et al., 2015). Consensus full-length (FL) transcripts generated from Iso-Seq data were aligned to the genome using Minimap2 (v2.24) and assembled using StringTie2 (v2.2.1; parameters: -L -m 150 -f 0.1 -t) (Li, 2018; Pertea et al., 2015).

For homology-based prediction, protein sequences of Cucurbitaceae downloaded from the UniProt database were aligned to the PKUMo genome using Miniprot (v0.12-165, r237; parameter: -G 50k) (Li, 2023, UniProt Consortium, 2015). TransDecoder (v3.0.0; https://github.com/TransDecoder/TransDecoder) was used to identify candidate open reading frames and predict protein sequences from transcript sets derived from protein alignments and FL transcript alignments.

For *ab initio* prediction, AUGUSTUS (v3.4.0; https://github.com/Gaius-Augustus/Augustus) and GeneMark-ETP (v4.38; https://github.com/gatech-genemark/GeneMark-ETP) were applied to the repeat-masked genome. AUGUSTUS predictions were based on a species-specific model trained using the autoAugTrain.pl Perl script within the AUGUSTUS package, together with a hints file generated by the blat2hints.pl Perl script.

All gene model evidence was integrated using EVidenceModeler (v2.1.0; https://github.com/EVidenceModeler/EVidenceModeler) to generate a non-redundant set of gene structures, with weights assigned according to the input sources. Two rounds of the PASA pipeline (v2.5.3; https://github.com/PASApipeline/PASApipeline) were performed to identify isoforms and untranslated regions by incorporating Iso-Seq and full-length cDNA sequences. Potential errors in gene structures were manually corrected by aligning short reads and Iso-Seq reads using IGV-sRNA (https://gitee.com/CJchen/IGV-sRNA). Functional annotation was conducted using eggNOG-mapper (v2.1.12), resulting in functional annotation of 95.68% of the predicted genes in PKUMo (Cantalapiedra et al., 2021).

### Variation between the PKUMo and HZAU genomes

SyRI was used to detect genomic variation between the PKUMo and HZAU genomes, including syntenic blocks, structural rearrangements (inversions, translocations, and duplications), local variations (SNPs, insertions/deletions [indels], and copy number variations [CNVs]) within these regions, and unaligned regions (Goel et al., 2019). Plotsr was subsequently used to visualize the identified syntenic regions and structural variations between the two genomes (Goel and Schneeberger, 2022).

### GO enrichment analysis

All GO-annotated gene entries from the eggNOG-mapper output were used as the background gene set, and PKUMo genes located in regions unaligned to the HZAU genome were defined as the target set for GO enrichment analysis. The analysis was conducted using the clusterProfiler R package with a significance cutoff of *p* < 0.05.

### Identification, classification, and phylogenetic analysis of LTR retrotransposons

LTR-RTs in the PKUMo genome were identified using LTRharvest and LTR_finder (Ellinghaus et al., 2008; Ou and Jiang, 2019), followed by integration with LTR_retriever to ensure accurate detection (Ou and Jiang, 2018). These LTR-RTs were classified using TEsorter through comparison with the REXdb-plant database (http://repeatexplorer.org/) (Neumann et al., 2019; Zhang et al., 2022a).

Based on the LTR-RT classification, reverse transcriptasedomain sequences from the Copia and Gypsy families were extracted. Phylogenetic analysis was performed using TEsorter package scripts, including sequence alignment with MAFFT (https://mafft.cbrc.jp/alignment/software/) and tree construction with IQ-TREE2 (Katoh et al., 2002; Minh et al., 2020).

### Identification and classification of duplicate genes

DupGen_finder was used to identify gene duplications and classify them into distinct types, including whole-genome duplicates, tandem duplicates, proximal duplicates, transposed duplicates, and dispersed duplicates (Qiao et al., 2019).

### Ks distribution of duplicated gene pairs

Protein and coding DNA sequences of duplicated gene pairs were extracted and analyzed using the ParaAT (v2.0) pipeline (Zhang et al., 2012), which performs sequence alignment and calculates synonymous substitution (Ks) values. The distribution of Ks values was visualized using custom R scripts.

### Mutant SNP calling

A total of 38 individuals from M_1_ materials and 30 individuals from M_2_ materials were randomly selected. All 68 samples were sequenced individually at a depth of 30×. Raw sequencing data were first processed using fastp (v0.23.4) for quality control to generate high-confidence clean reads, which were then aligned to the PKUMo reference genome using BWA-MEM (v0.7.17) with default parameters. Subsequently, GATK (v4.6.2.0) was used for variant calling to identify genomic variations from the alignment files (McKenna et al., 2010). After variant detection, SNPs were extracted and filtered based on quality thresholds (QD < 2.0, FS > 60.0, MQ < 40, SOR > 3, MQRankSum < −12.5, and ReadPosRankSum < −8.0). Finally, VCFtools (v0.1.16) with the --TsTv-summary parameter was used to conduct comprehensive classification of SNP subtypes, including transitions and transversions.

### EMS pollen treatment dosage and duration

Pollen grains were subjected to EMS treatment as modified from the method described by Deng et al. (2022). An adequate number of anthers were harvested from male flowers at full bloom in the early morning. For EMS treatment, the anthers were immersed in 5 ml of mineral oil containing the appropriate volume of EMS. A 1:15 suspension of EMS (Sigma M0880-5G) in mineral oil (Sigma M8410-1L) was first prepared and subsequently diluted to final concentrations of 0%, 0.1%, 0.15%, and 0.2% (v/v). After stirring with a glass rod for 5 min, the anthers and debris were removed using a fine-mesh kitchen strainer. The pollen–EMS mixture was then transferred to a 50 ml glass bottle with a tightly fitting lid, and the pollen grains were gently stirred for the indicated durations (0, 40, 60, and 80 min) using a magnetic stirrer.

Pollen grains treated with EMS were cultured on a medium containing 1% (w/v) agar, 2 g sucrose, 20 mg H_3_BO_3_, 41.6 mg Ca(NO_3_)^2^, 21.7 mg MgSO_4_·7H_2_O, and 10 mg KNO_3_ in 1000 mL of distilled water, adjusted to pH 8.0. Aliquots of pollen samples in mineral oil (10 μl) were dispensed into 100 μl of germination solution in a 48-well tissue culture plate, with each well serving as one replicate per treatment. The samples were left uncovered but loosely capped to allow gas exchange. After 2 h of incubation, the percentage of germinated pollen grains was recorded. A pollen grain was considered germinated when the length of the pollen tube exceeded twice the width of the pollen grain. For each replicate, 50 pollen grains were randomly evaluated. The germination rate of pollen treated with 0.1% EMS for 60 min was approximately 50%, corresponding to the median lethal dose; these conditions were therefore selected for subsequent experiments.

### Large-scale EMS treatment and phenotypic evaluation of the mutant population

A pollination experiment was conducted on 1000 PKUMo plants using EMS-treated pollen grains. The procedure was as follows: pollen grains were immersed in EMS solution, stirred with a magnetic stirrer for 40 min, and then allowed to stand for 5 min. The supernatant was subsequently decanted and discarded, and the remaining solution was filtered through filter paper to retain the treated pollen grains. A long-handled brush was used to evenly apply the treated pollen grains to the stigmas, completing the pollination process. The entire pollination operation was completed within 30 min after mutagenesis, and appropriate protective equipment was worn throughout to ensure safety. Finally, approximately 60,000 M_1_ seeds were harvested in the spring of 2021.

In the autumn of 2021, a total of 1000 M_1_ plants and 50 wild-type plants were grown. All M_1_ plants were self-pollinated, where conditions permitted, to generate M_2_ families. In the spring and fall of 2022 and the spring of 2023, a total of 800 M_2_ families were sown, with each family containing 24 seeds, and grown in plastic greenhouses. According to the method described by García et al. (2018), systematic phenotypic evaluations were conducted for all mutants exhibiting visible alterations throughout the growth cycle. For M_2_ plants that exhibited male-sterile flowers, mutants were maintained by crossing with wild-type plants, and their genetic characteristics were analyzed. All M_2_ plants were self-pollinated to generate M_3_ families. For each M_3_ family carrying recessive single-gene mutations, 24 seeds were sown in plastic greenhouses in the autumn of 2023 for further evaluation. Field management was carried out in accordance with recommended standards for commercial production, including seedling cultivation, fertilization, irrigation, and disease control.

### Genetic analysis of mutations

The χ^2^ test was used to evaluate whether the segregation ratios of each mutant phenotype in the M_2_ generation conformed to expected genetic ratios. Additionally, when two distinct mutant phenotypes were observed within the same family, the χ^2^ test was applied to assess the independent segregation of the corresponding mutant alleles. The probability of a type II error in single-gene inheritance was set at 10%.

### Number of plants required to construct a saturated butternut squash mutant library

The number of mutant plants required to construct a saturated butternut squash mutation library was estimated. Probabilities were calculated using the following formula (Krysan et al., 1999): *P* = 1 − (1 − [*L*/*C*])^*nf*^, where *P* represents the probability of identifying at least one functional mutation in a given gene, *L* is the average gene length (kb), *C* is the haploid genome size, *n* is the number of independent mutants, and *f* is the average number of mutations per mutant. In this study, *L*/*C* was approximated using the total gene number of the PKUMo genome. This calculation assumes that mutations occur randomly across the genome.

### Bulk segregant analysis

For the dwarf and chlorotic traits, two extreme phenotype bulks were constructed, each comprising 30 individuals from the corresponding F_2_ populations. These pools were subjected to next-generation sequencing (NGS) at an average depth of ∼30× per pool. The parental lines (male and female) were also sequenced by NGS at an average depth of 20×. Raw reads were quality-controlled using fastp (v0.23.4) to remove low-quality reads. High-quality clean reads were then aligned to the reference genome using BWA-MEM (v0.7.17) with default parameters. Variant calling was performed following GATK (v4.6.2.0; McKenna et al., 2010) best practices with stringent filtering criteria: SNPs were retained if they met MQ ≥ 40, FS ≤ 60, QD ≥ 2, SOR ≤ 3, MQRankSum ≥ −12.5, and ReadPosRankSum ≥ −8.0. Indels and non-biallelic sites were excluded to obtain high-confidence biallelic SNPs. These filtered SNPs were used for BSA via QTL-seq (v2.2.9; Sugihara et al., 2022). The Δ(SNP-index) was calculated, and candidate genomic regions associated with the mutant phenotype were identified using a sliding window approach (window size = 200 kb; step size = 10 kb).

### Identification of candidate genes for two selected mutants

To identify sequence variants in the candidate gene of the yellow-leaf mutant, the exons and introns of the candidate gene were amplified from the corresponding mutant materials. PCR was performed following the protocol of Phanta Max Master Mix (Vazyme, Nanjing, China) using sequencing primers. The reaction mixture consisted of 1× Phanta Max Master Mix, 10 μM of each primer, 50 ng of genomic DNA, and ddH_2_O added to a final volume of 50 μl. The PCR program included an initial denaturation at 95°C for 3 min, followed by 37 cycles of 94°C for 30 s, 55°C for 30 s, and 72°C for 3 min. PCR products were excised from agarose gels and purified using the Agarose Gel DNA Column Recovery Kit (TIANGEN, Beijing, China), followed by Sanger sequencing (Tsingke, Qingdao, China). Sequence chromatograms from wild-type and mutant samples were analyzed using SnapGene software. Mapping of the micro mutant was performed using the same approach as for the yellow-leaf mutants.

### RNA-seq analysis

Total RNA was isolated from leaf tissues of 30-day-old PKUMo yellow-leaf mutant and wild type seedlings using Freezol Reagent (Vazyme, Nanjing, China). RNA-seq libraries were constructed using the Vazyme Library Prep Kit and sequenced on the Illumina platform by Novogene (Tianjin, China). Bioinformatic analyses included quality control with fastp, alignment to the *C. moschata* PKUMo reference genome using HISAT2 (v2.2.1), read quantification with featureCounts, and identification of differentially expressed genes (DEGs) using DESeq2. Subsequent GO enrichment analysis was performed using the R package clusterProfiler (Yu et al., 2012; Liao et al., 2014; Love et al., 2014).

## Data availability

All raw sequencing data generated in this study have been deposited in the Genome Warehouse of the National Genomics Data Center, Beijing Institute of Genomics, Chinese Academy of Sciences, under BioProject accession number PRJCA025343. All datasets and mutant phenotype data are available through the PumpkinDB website (http://omicsplant.cn/Cucurbitamoschata/). All materials used in this study are available upon request.

Full mutant-related datasets are publicly available for free browsing and download. Users can retrieve specific mutant information by entering a mutant ID, trait type, or associated candidate gene name. For requests for viable mutant seeds or seedlings, please contact the corresponding author, Xingping Zhang, with a formal application outlining the research purpose, experimental design, and intended use. Material distribution will be processed promptly upon the signing of a Material Transfer Agreement (MTA).

The data supporting the findings of this study are included within the article and its supplemental information.

## Funding

This work was supported by the Provincial Technology Innovation Program of Shandong, the Ningbo Science and Technology Innovation Project (2021Z132), and the Weifang Seed Innovation Group.

## Acknowledgments

The authors thank all laboratory members for their technical assistance and valuable discussions during the research and manuscript preparation. The authors declare no competing interests.

## Author contributions

X.Z., Y.D., H.H., X.W.D., and Y.P. conceived and supervised the study. J.L., Z.C., and K.L. wrote the manuscript. K.L. and Z.C. performed the bioinformatics analyses. K.L. constructed the database. J.T. performed the ChIP-seq experiments. J.L., J.S., and Y.D. conducted the field experiments. J.S. carried out sampling and molecular laboratory experiments. X.Z., H.H., Y.D., K.L., and Y.P. contributed to the discussion and revision of the manuscript. All authors read and approved the final manuscript.

## Supplemental information

Document S1. Supplemental Figures 1–16 and Supplemental Tables 1–18

Document S2. Article plus supplemental information
