# Supplementary material for: Telomere-to-telomere genome assembly and a mutant library empower functional genomics and genetic improvement in Cucurbita moschata
Source: Plant Commun. 2026 Mar 25;7(5):101836. doi: 10.1016/j.xplc.2026.101836 (PMC13174253; doi:10.1016/j.xplc.2026.101836)
Supplement: Document S1. Supplemental Figures 1–16 and Supplemental Tables 1–18 [file mmc1.pdf]

Plant Communications, Volume *7*

Supplemental information

Telomere-to-telomere genome assembly and a mutant library empower functional genomics and genetic improvement in *Cucurbita moschata*

Juan Li, Zenghui Chen, Kui Li, Jingsheng Tan, Jing Sun, Xing Wang Deng, Younghoon Park, Hang He, Yun Deng, and Xingping Zhang

Telomere-to-telomere genome and mutant library empower functional genomics and genetic improvement in *Cucurbita moschata*

Juan Li[1,2,4], Zenghui Chen[2,4], Kui Li [2,4], Jingsheng Tan[2], Jing Sun[2], Xing Wang Deng[2], Younghoon Park[1,3] *, Hang He[2, *], Yun Deng[2, *], Xingping Zhang[2, *]

[1]Department of Horticultural Bioscience, Pusan National University, Miryang 50463, Republic of Korea

[2]Peking University Institute of Advanced Agricultural Sciences, Shandong Laboratory of Advanced Agricultural Sciences at Weifang, Weifang, Shandong 261000, China

[3]Life and Industry Convergence Research Institute, Pusan National University, Miryang 50463, Republic of Korea

[4]Authors contributed equally

*Corresponding authors. E-mail: ypark@pusan.ac.kr;hang.he@pku-iaas.edu.cn; yun.deng@pku-iaas.edu.cn; xingping.zhang@pku-iaas.edu.cn

Short summary: This study reports a gap-free, telomere-to-telomere genome assembly of *Cucurbita moschata* PKUMo, along with an analysis of structural variations in comparison to the closely related *Cucurbita maxima* genome. A mutant library of 60,000 M_1 seeds and 800 M_2 families, demonstrating 15.5% visible phenotypic variation was generated using pollen EMS mutagenesis. The T2T genome and the mutant library provide valuable resources for gene discovery and molecular breeding within Cucurbita species.

Supplementary Figures

A

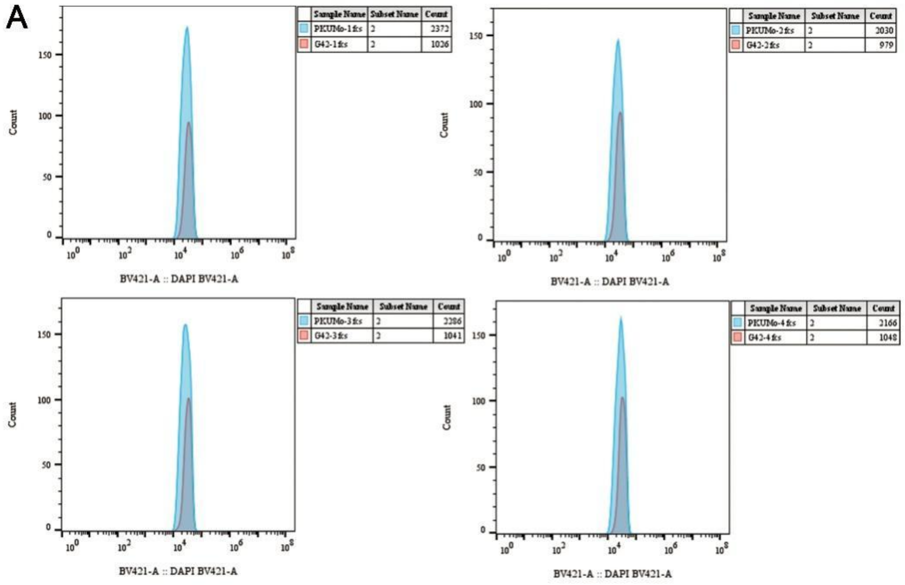

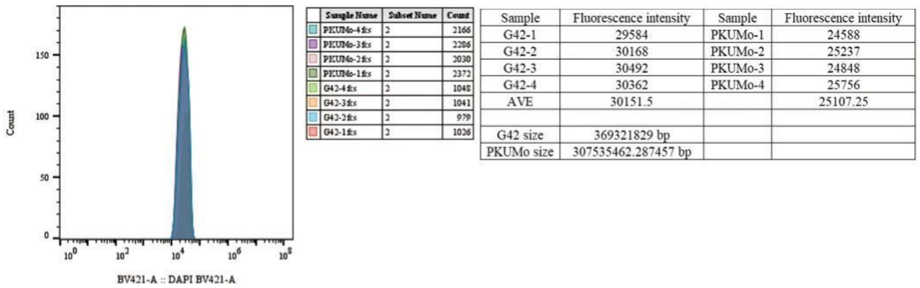

Sample	Fluorescence intensity	Sample	Fluorescence intensity
G42-1	29584	PKUMo-1	24588
G42-2	30168	PKUMo-2	25237
G42-3	30492	PKUMo-3	24848
G42-4	30362	PKUMo-4	25756
AVE	30151.5		25107.25
G42 size	369321829 bp		
PKUMo size	307535462.287457 bp		

B

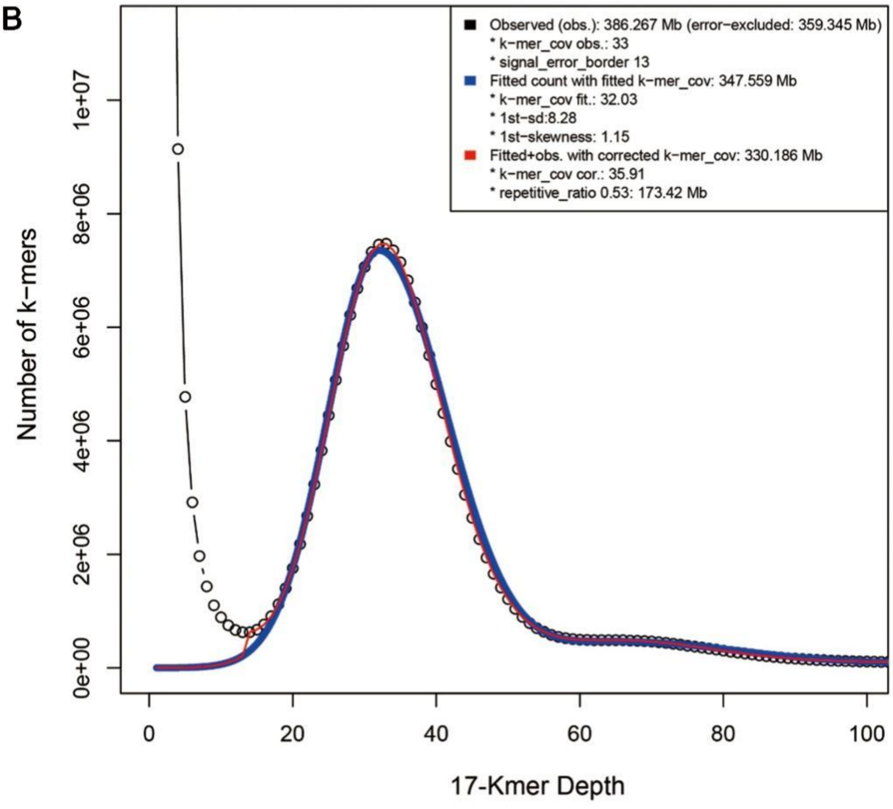

Supplementary Figure 1. Flow cytometry analysis and distribution of 17-mer frequency in the PKUMo genome. (A) Flow cytometry analysis of PKUMo and G42 genome. Flow cytometry results of four replicates and summaries of PKUMo and G42 stained with DAPI. Based on the fluorescence intensity of G42 and the genome size, the genome size of PKUMo is estimated to be 307,535,462 bp. (B) Distribution of 17-mer frequency in the PKUMo genome. The horizontal axis represents k-mer depth. The black line depicts the observed k-mer frequency, while the blue line corresponds to the fitted model. Notably, the red line represents the refined fitted model incorporating k-mer corrections, which serves as the key reference for genome size estimation. Based on the distinct k-mer volume peak at 35.91, the genome size was estimated to be 330.18 Mb.

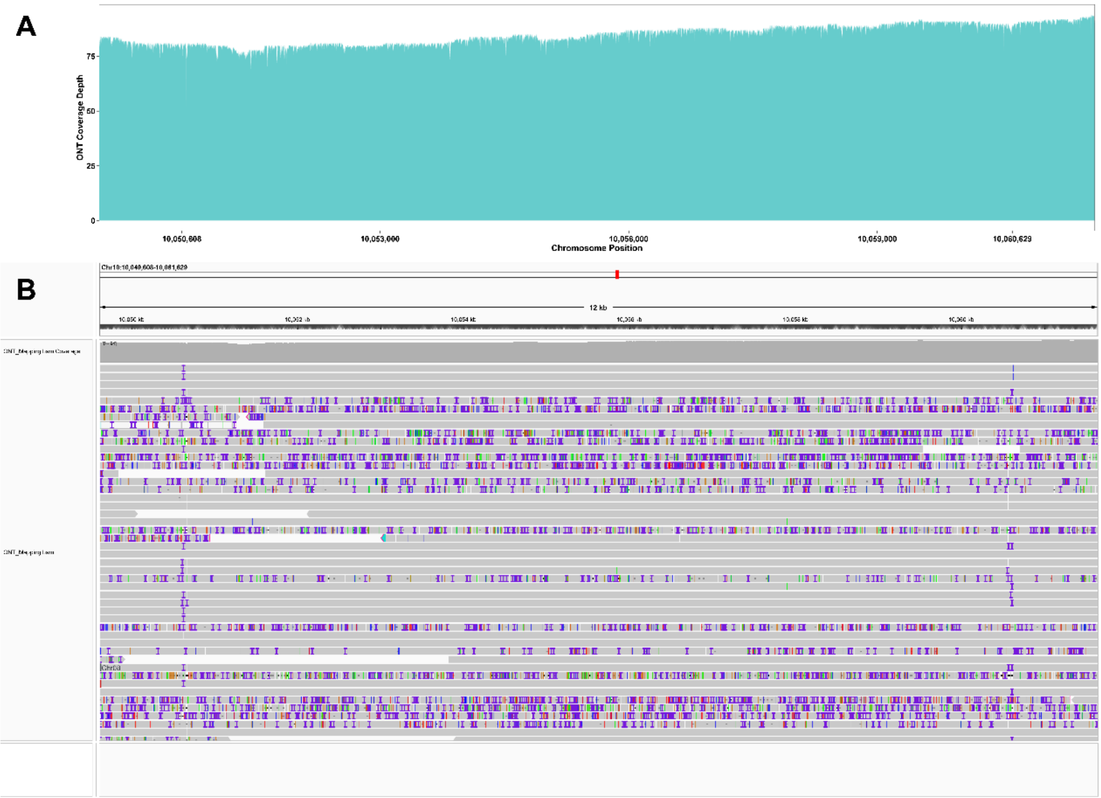

Supplementary Figure 2. Validation of the gap on Chr10 (10050608–10060629) using ONT read mapping. (A) ONT reads exhibit high and uniform mapping depth across the gap region. (B) IGV visualization of read mappings indicates no breakpoints within the gap region.

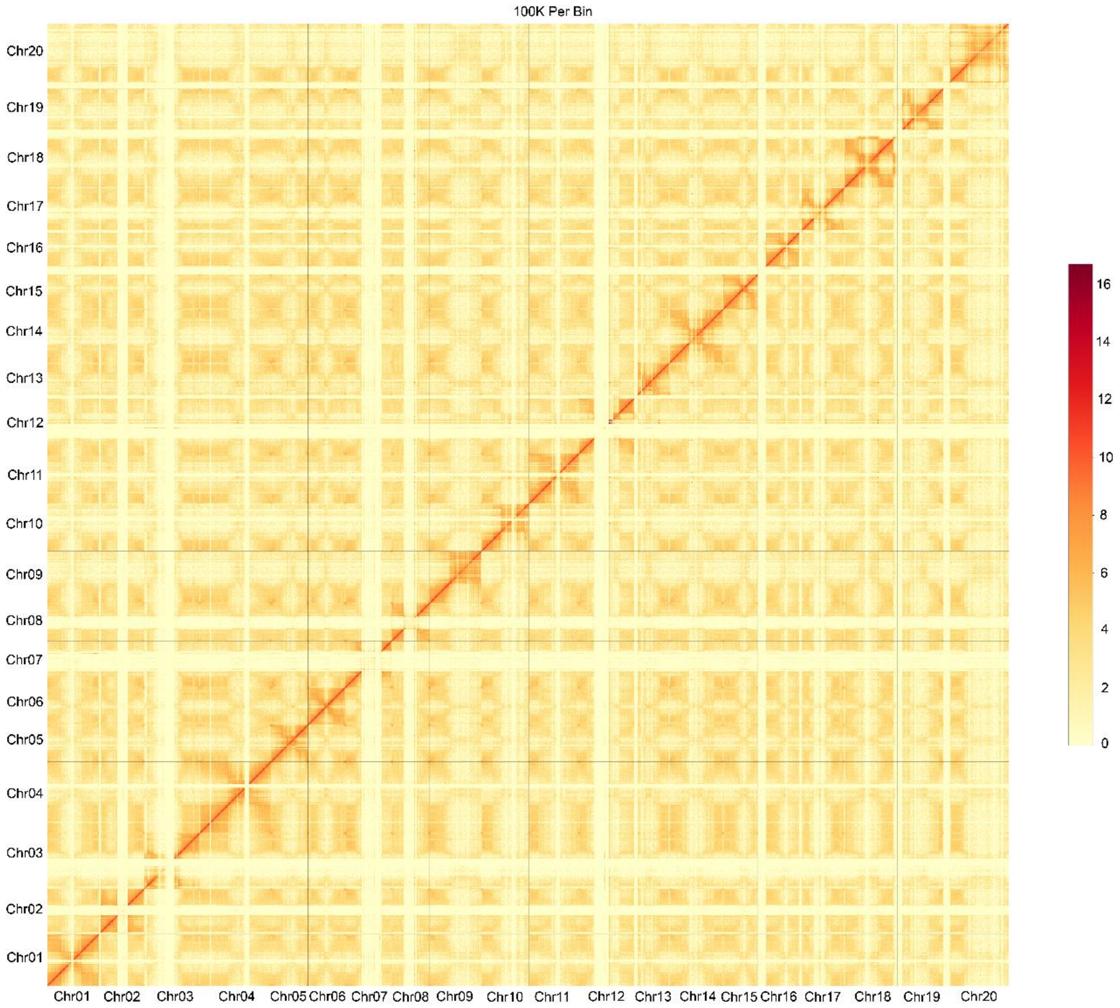

Supplementary Figure 3. Genome-wide Hi-C interaction map of the PKUMo genome assembly at a resolution of 100 kb.

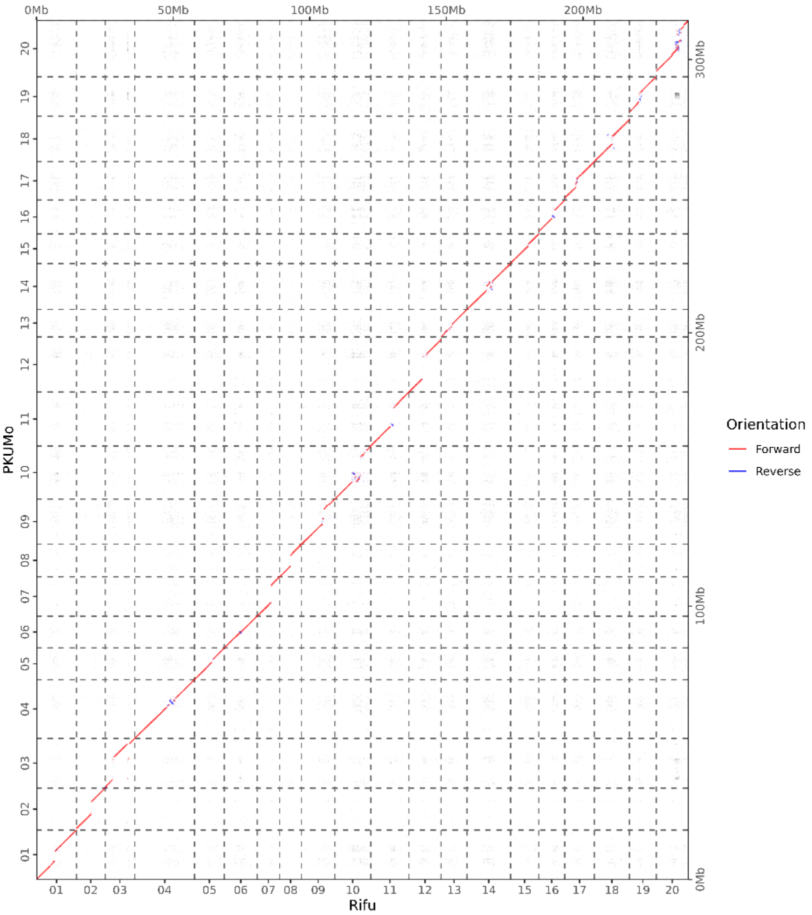

Supplementary Figure 4. Collinearity analysis between the PKUMo and Rifu (*Cucurbita moschata*) genomes.

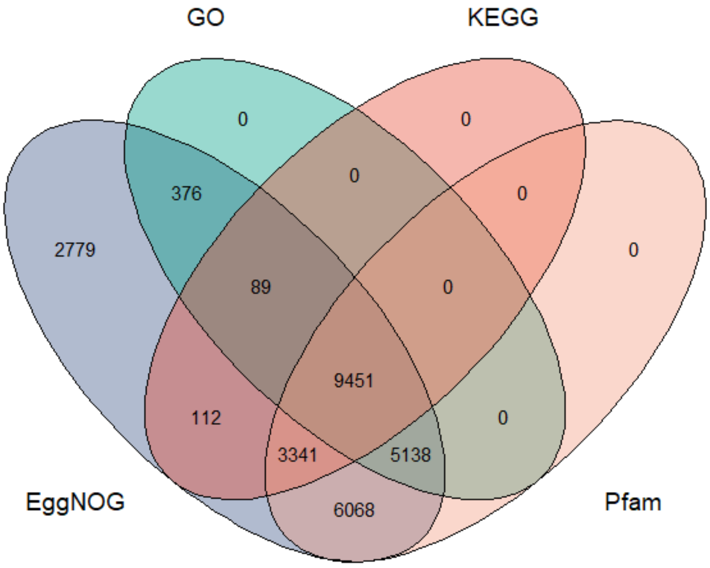

Supplementary Figure 5. Number of PKUMo genes annotated in the Gene Ontology (GO), Kyoto Encyclopedia of Genes and Genomes (KEGG), and Pfam databases.

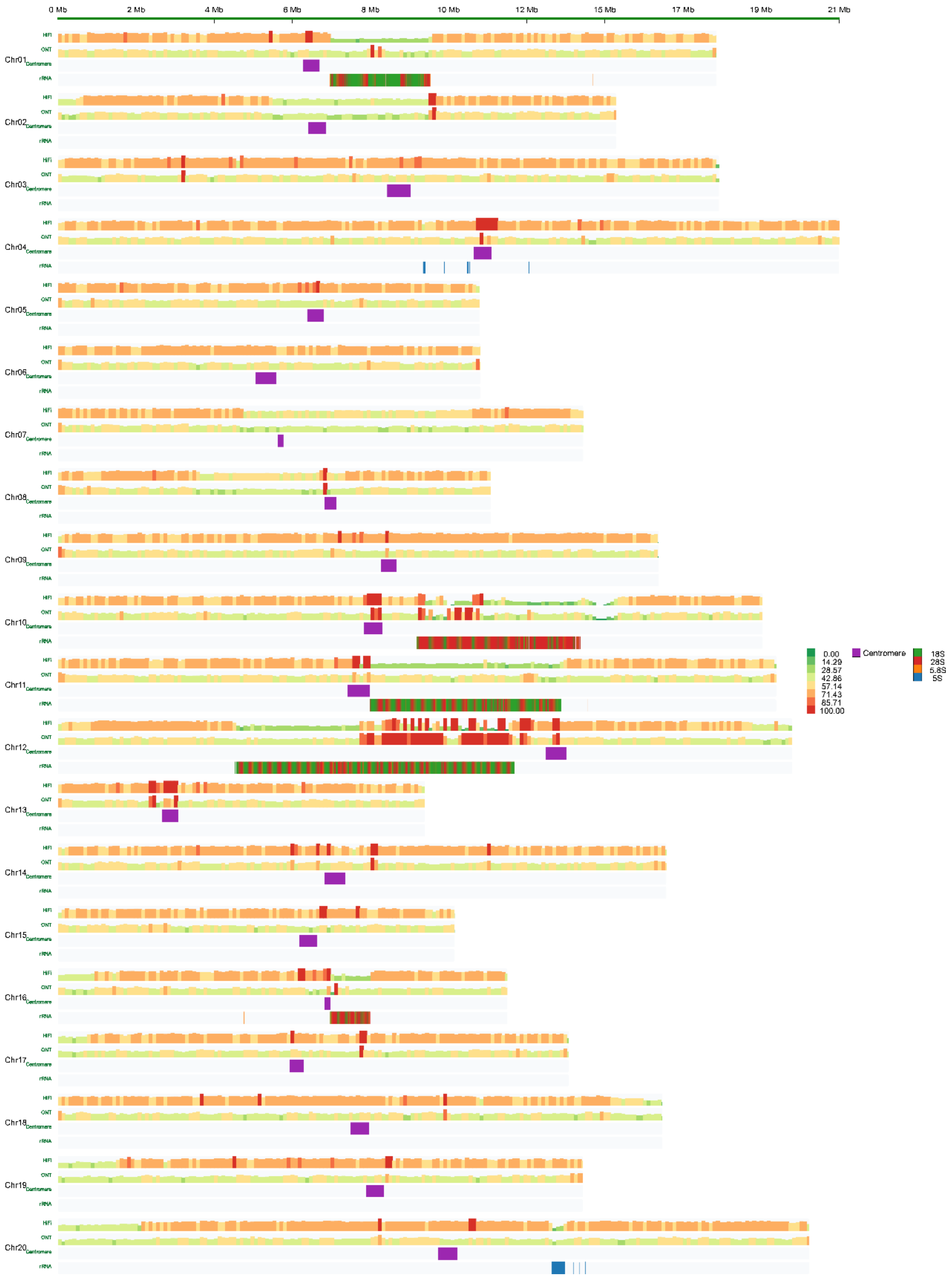

Supplementary Figure 6. Reads mapping, centromeric and rDNA characterization of the PKUMo genome. For each chromosome, the tracks from top to bottom represent HiFi sequencing read coverage, ONT sequencing read coverage, centromeric region, and rDNA distribution across the chromosome, respectively.

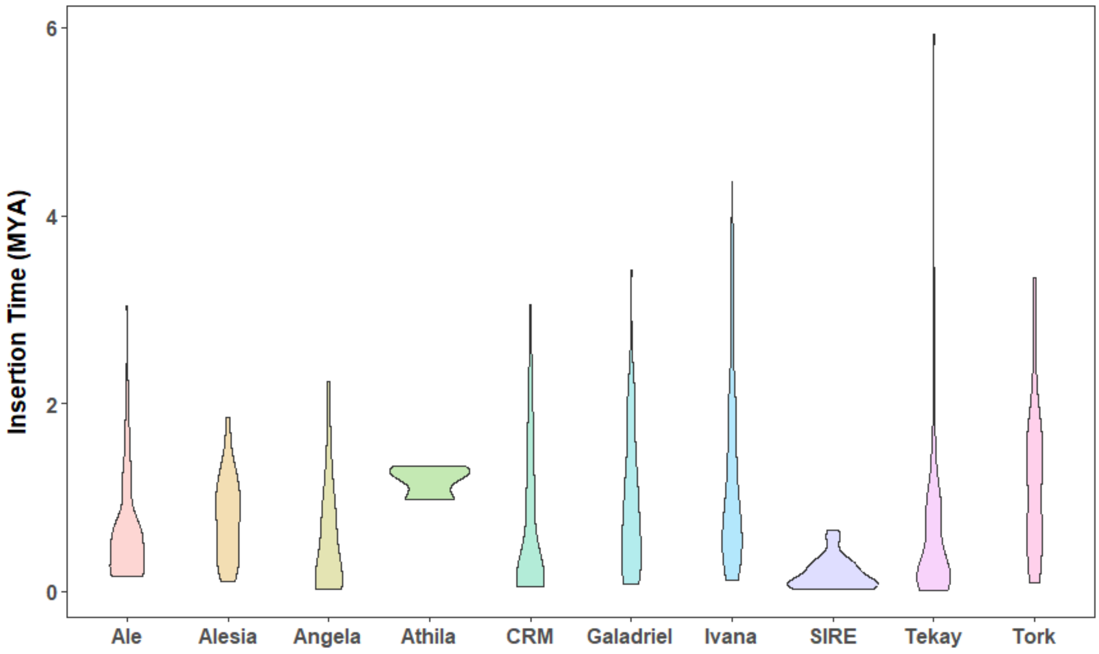

Supplementary Figure 7. Distribution of LTR - RT insertion times across clades of the *Copia* and *Gypsy* superfamilies.

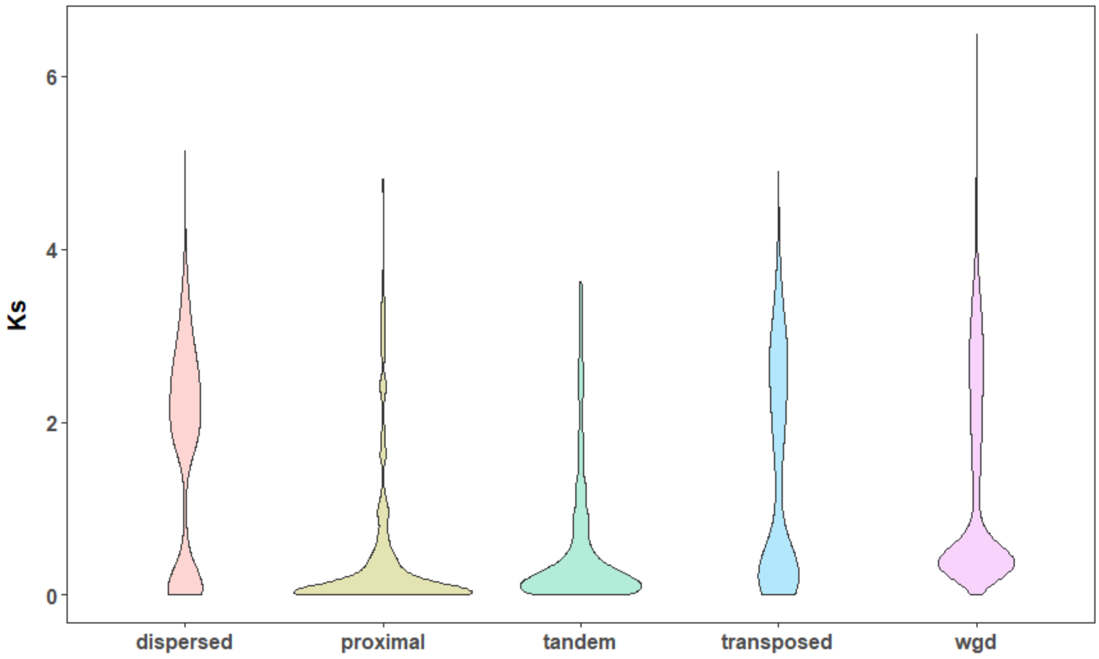

Supplementary Figure 8. Distribution of Ks values for gene pairs derived from five distinct duplication modes.

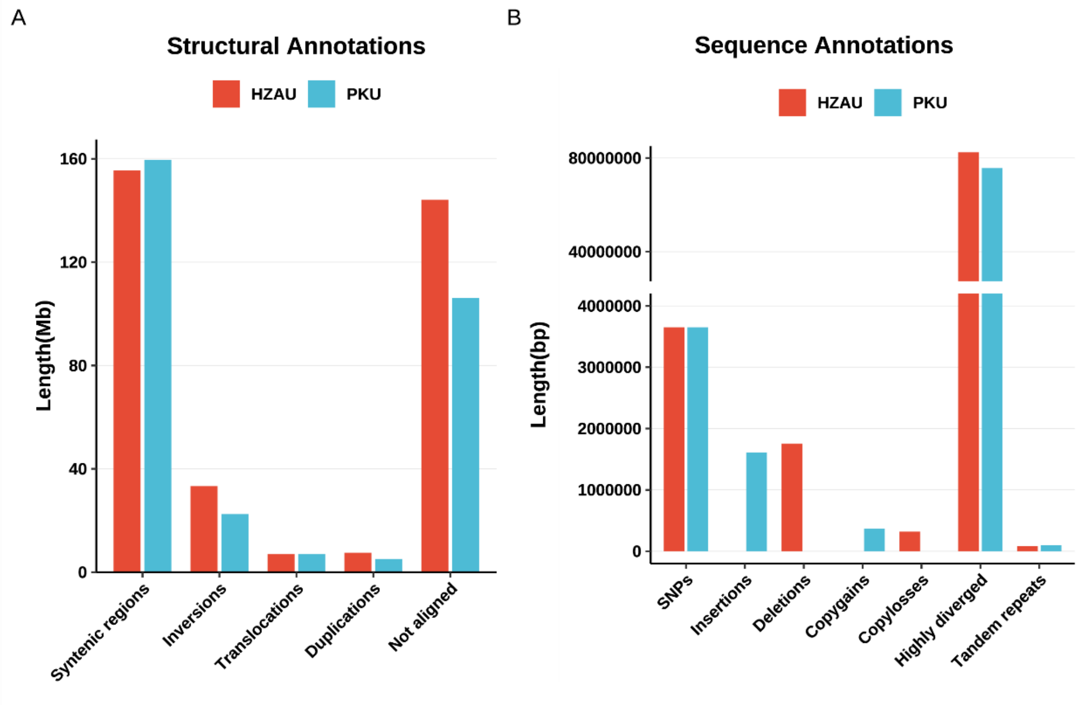

Supplementary Figure 9. Genomic variations between the PKUMo (*Cucurbita moschata*) and HZAU (*Cucurbita maxima*) genomes. (A) Lengths of structural variations in the PKUMo and HZAU genomes. (B) Lengths of local variations in the PKUMo and HZAU genomes.

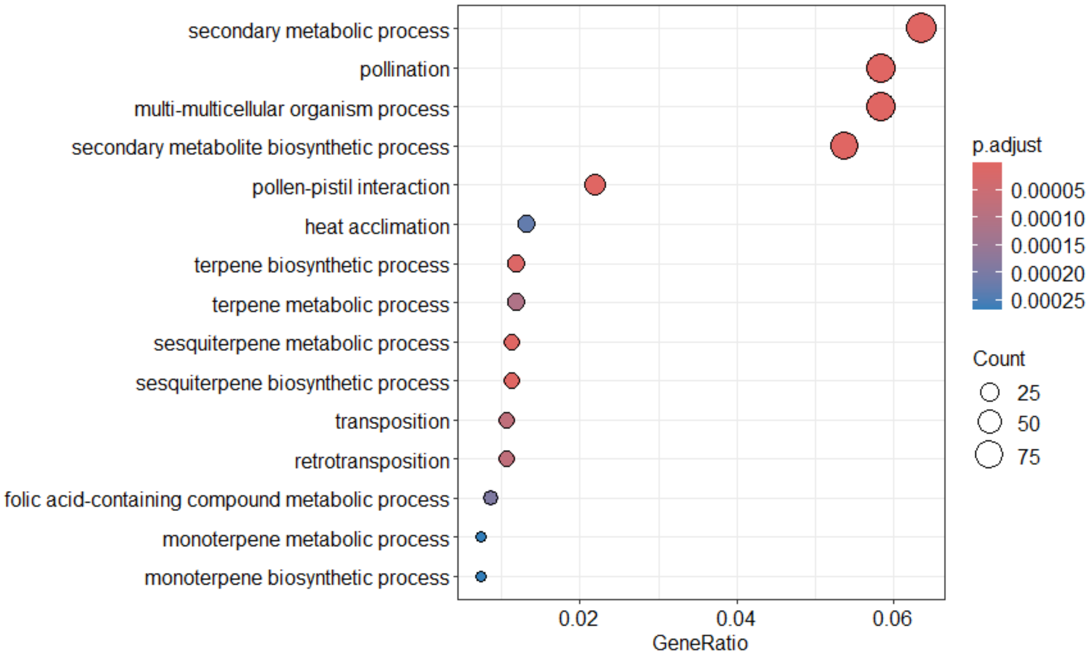

Supplementary Figure 10. GO enrichment analysis of genes in the unaligned regions between the PKUMo (*Cucurbita moschata*) and HZAU (*Cucurbita maxima*) genomes.

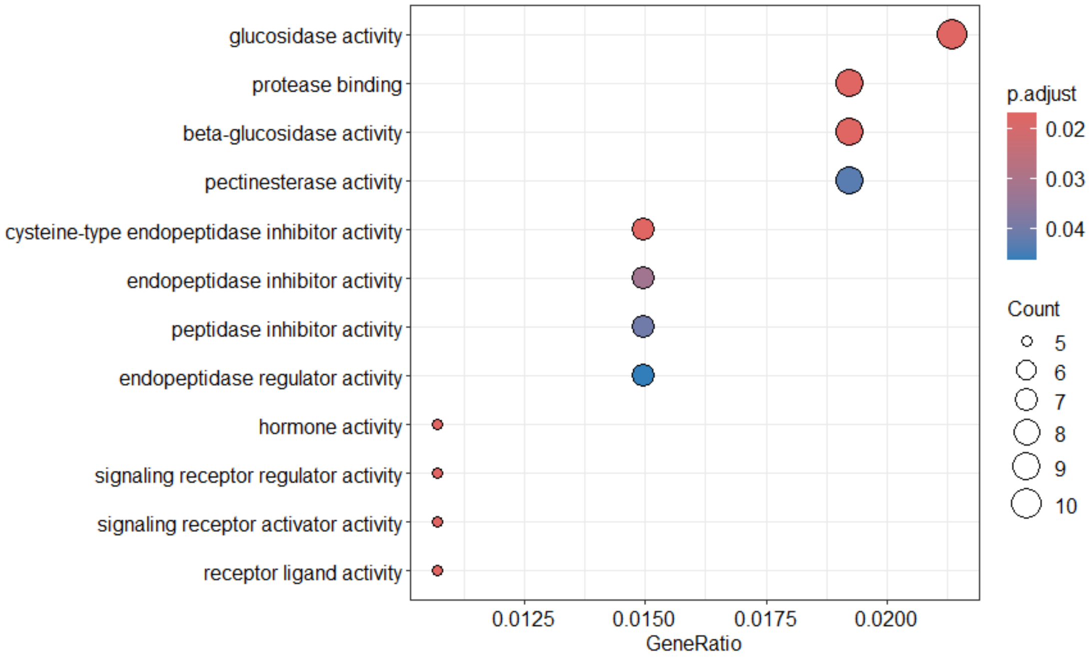

Supplementary Figure 11. GO enrichment analysis of genes in the inversion regions between the PKUMo (*Cucurbita moschata*) and HZAU (*Cucurbita maxima*) genomes.

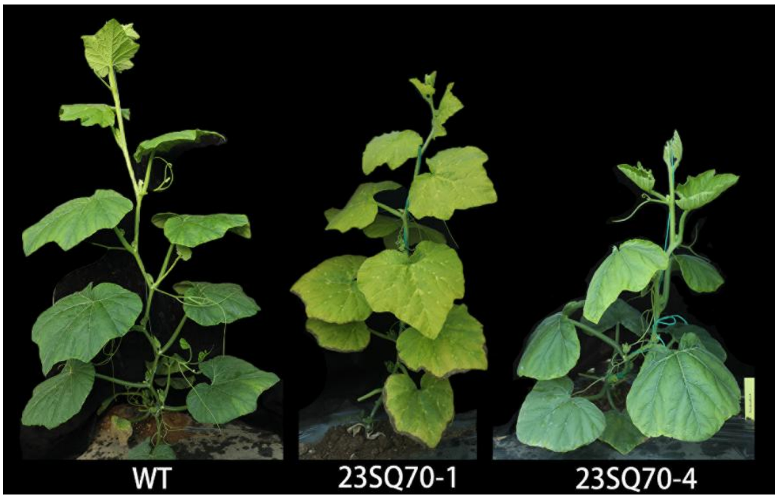

Supplementary Figure 12. Multi-mutation line 23SQ070. There are two types of mutations in the 23SQ70 line: chlorosis (23SQ70-1) and dwarfism (23SQ70-4).

A

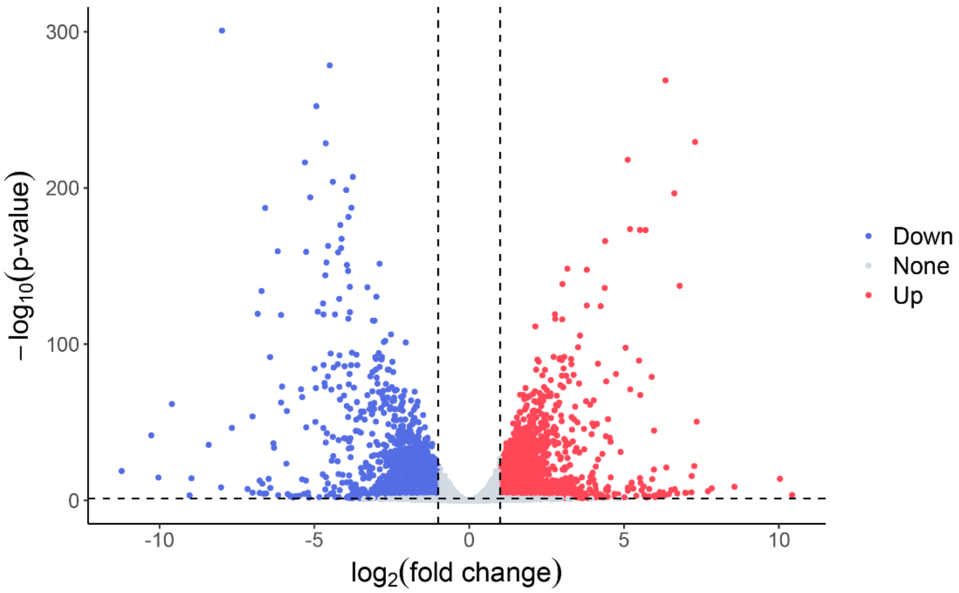

B

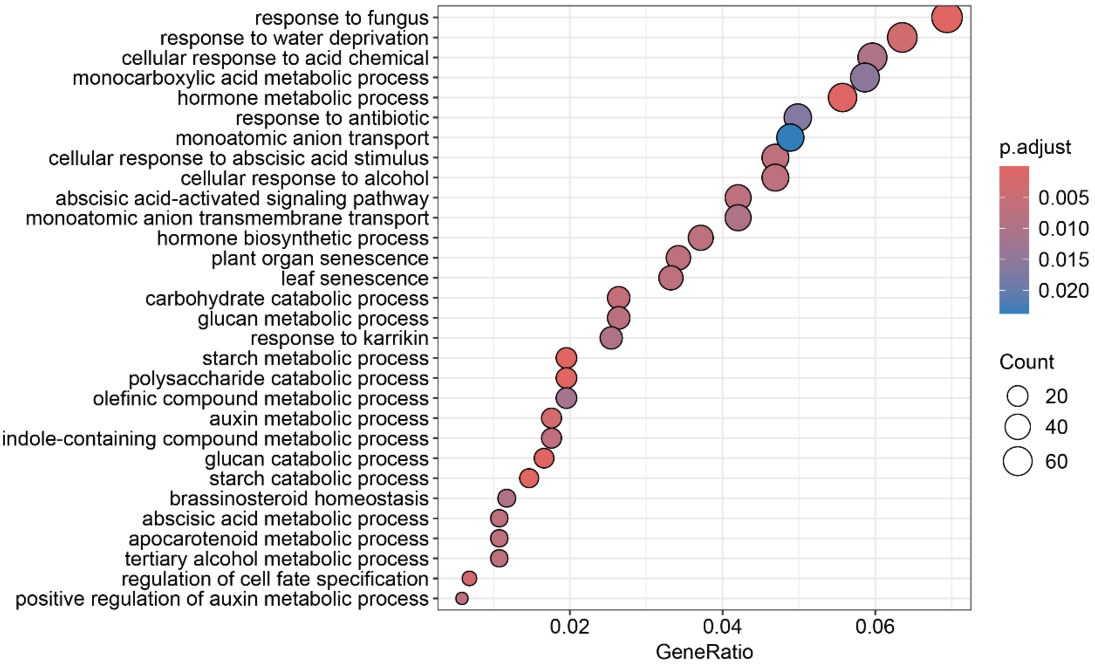

Supplementary Figure 13. Transcriptome-wide identification of significantly up- and down-regulated genes between the yellow and green group of PKUMo.

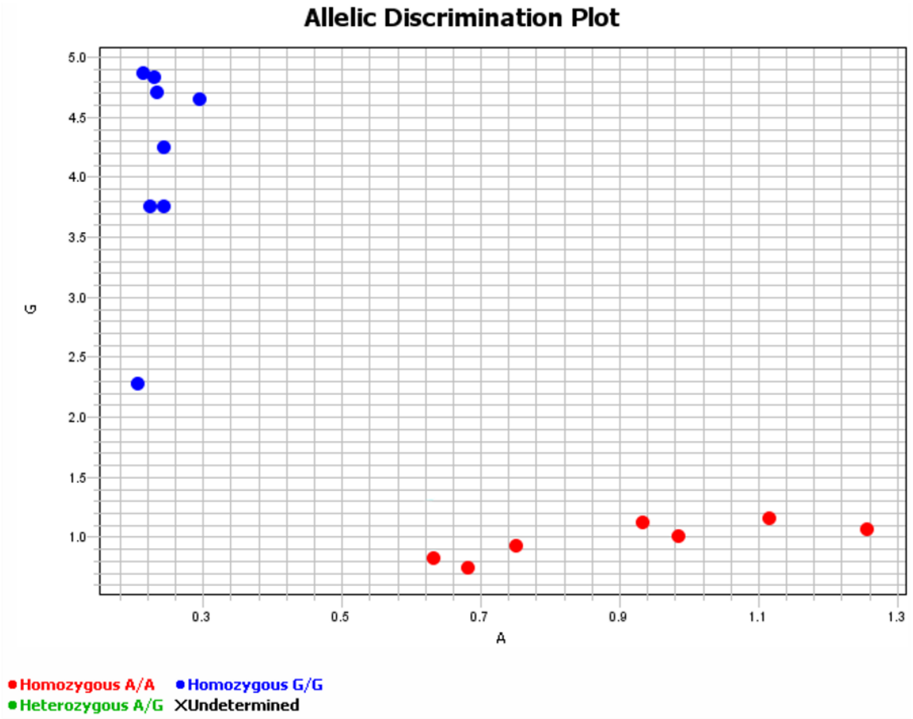

Supplementary Figure 14. Genotype of wild type and yellow mutant.

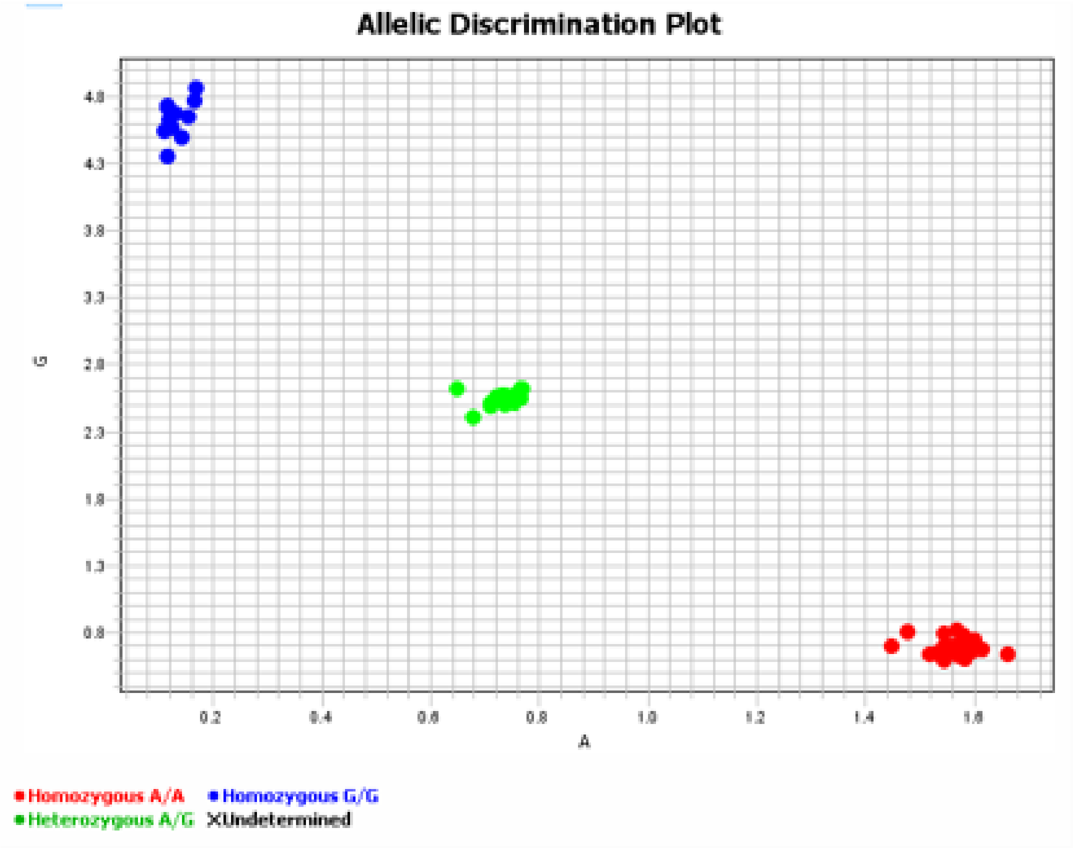

Supplementary Figure 15. Genotyping of m15 × WT F_2 population using KASP markers. Red, green, and blue dots represent homozygous mutants, heterozygous mutants, and wild-type individuals, respectively. KASP primer sequences: F1: GAAGGTGACCAAGTTCATGCTCATCGTCTCTGATGAAGTCGTCTCG; F2: GAAGGTCGGAGTCAACGGATTCATCGTCTCTGATGAAGTCGTCTCA; R: TCCAACGGCTCTAGAGCAACCT.

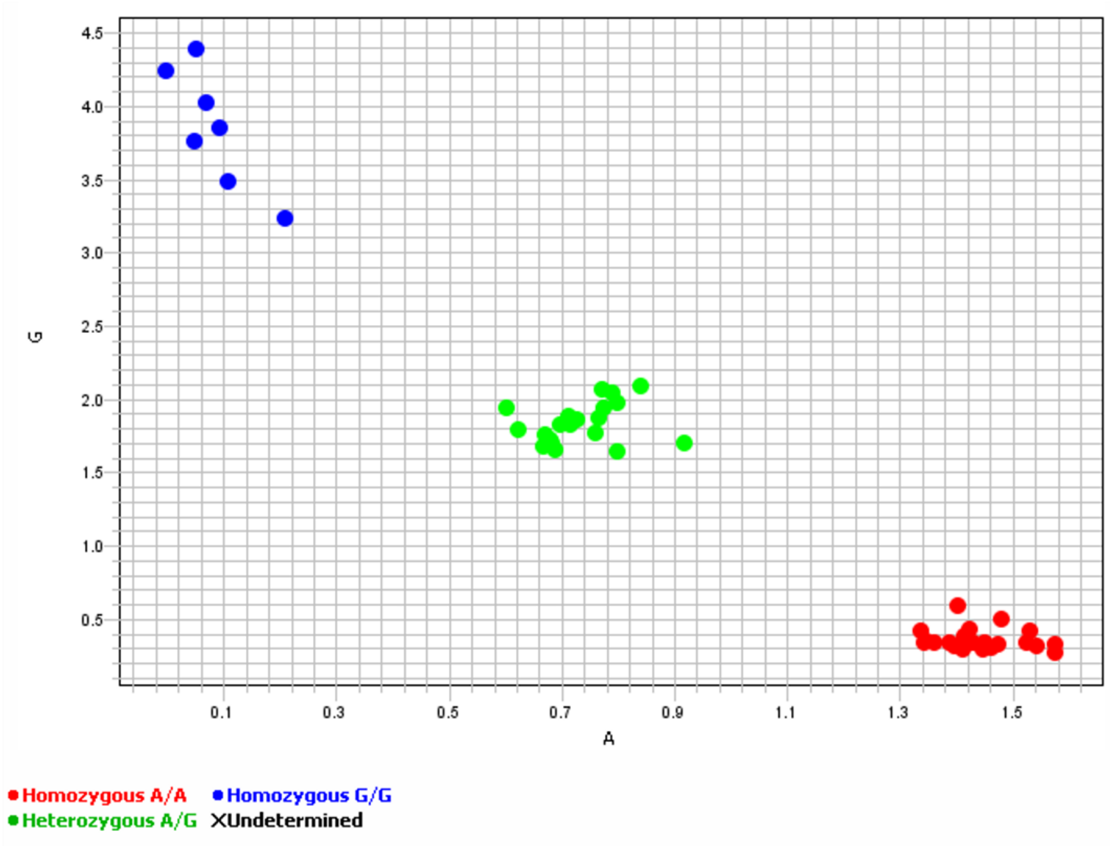

Supplementary Figure 16. Genotyping of mSq × WT F_2 population using KASP markers. Red, green, and blue dots represent homozygous mutants, heterozygous mutants, and wild-type individuals, respectively. KASP primer sequences: F1: GAAGGTGACCAAGTTCATGCTTGTTCCCACCGAACGGATTTCAGG; F2: GAAGGTCGGAGTCAACGGATTTGTTCCCACCGAACGGATTTCAGA; R: ACGCATCAGAAATGAGGATATCAAG.

Supplementary Tables

Supplementary Table 1. Sequencing data statistics of the PKUMo genome.

Library type	Total data (G)	Read N50 (bp)	Sequence coverage
HiFi	27.34	14,741	83.43
ONT ultra-long	25.95	72,250	80.65
Hi-C	76.60	-	246.30
RNA-Seq	487.33	150	
ISO-Seq	1.78	2,783	

Supplementary Table 2. Statistic of initially genome assembly.

Sample ID	Initial assembly		Purge haplotigs	
	Contig(bp)	Number	Contig(bp)	Number
Total	329409519	68	312384757	21
Max	21479097	-	21479097	-
Number ≥2000	-	68	-	21
N50	16503632	9	16503632	9
N60	14440264	11	14440264	11
N70	12103242	14	14025994	13
N80	11601324	17	11905199	15
N90	10081957	20	10905709	18

Supplementary Table 3. Identification of telomeres on the PKUMo genome.

Chr	Length	Leftnum	Rightnum
Chr01	18,106,230	1,778	1,266
Chr02	15,350,078	1,663	1,901
Chr03	18,177,716	3,947	7,476
Chr04	21,483,489	1,335	1,739
Chr05	11,604,023	1,238	2,476
Chr06	11,612,697	1,483	1,483
Chr07	14,442,624	1,924	1,503
Chr08	11,907,414	1,158	1,927
Chr09	16,519,219	2,142	2,962
Chr10	19,373,740	1,804	1,574
Chr11	19,751,869	1,257	2,525
Chr12	20,189,906	3,295	1,602
Chr13	10,084,761	1,942	1,182
Chr14	16,729,065	1,167	1,586
Chr15	10,908,248	1,895	1,490
Chr16	12,357,011	1,061	2,323
Chr17	14,039,970	1,440	2,550
Chr18	16,617,563	1,630	1,988
Chr19	14,432,088	1,221	1,400
Chr20	20,656,156	1,190	1,752

Supplementary Table 4. Statistics of contigs anchored to each chromosome of the PKUMo genome.

Chr	Anchored contigs	Length(bp)
Chr01	1	18,106,230
Chr02	1	15,350,078
Chr03	1	18,177,716
Chr04	1	21,483,489
Chr05	1	11,604,023
Chr06	1	11,612,697
Chr07	1	14,442,624
Chr08	1	11,907,414
Chr09	1	16,519,219
Chr10	2	19,373,740
Chr11	1	19,751,869
Chr12	1	17,233,258
Chr13	1	10,084,761
Chr14	1	16,729,065
Chr15	1	10,908,248
Chr16	1	12,357,011
Chr17	1	14,039,970
Chr18	1	16,617,563
Chr19	1	14,432,088
Chr20	1	20,656,156

Supplementary Table 5. Mapping statistics of HiFi and ONT reads to the PKUMo genome (314,343,867 bp).

Type	Average sequencing depth	Total reads	Mapped reads	Mapping rate (%)	CoverageBase(bp)	Coverage (%)
HiFi	83.43	4795000	4784580	99.78	313632749	99.77
ONT	80.65	1639211	1637204	99.88	314050355	99.91

Supplementary Table 6. Genome assembly completeness assessment of PKUMo using Benchmarking Universal Single-Copy Orthologs (BUSCO).

Type	Number	Percentage (%)
Complete BUSCOs	1589	98.5
Complete and single-copy BUSCOs	1342	83.1
Complete and duplicated BUSCOs	247	15.3
Fragmented BUSCOs	13	0.8
Missing BUSCOs	12	0.7
Total BUSCO groups searched	1614	-

Supplementary Table 7. Chromosome length comparison between *C. moschata* Rifu and PKUMo genome.

Chr	Rifu	PKUMo	Novel sequence (bp)
Chr01	14,627,692	18,106,230	3,478,538
Chr02	10,523,820	15,350,078	4,826,258
Chr03	10,828,782	18,177,716	7,348,934
Chr04	21,859,252	21,483,489	0
Chr05	10,947,195	11,604,023	656,828
Chr06	11,971,590	11,612,697	0
Chr07	8,233,962	14,442,624	6,208,662
Chr08	8,001,254	11,907,414	3,906,160
Chr09	12,190,726	16,519,219	4,328,493
Chr10	13,139,847	19,373,740	6,233,893
Chr11	14,005,585	19,751,869	5,746,284
Chr12	11,750,945	20,189,906	8,438,961
Chr13	9,526,514	10,084,761	558,247
Chr14	15,889,863	16,729,065	839,202
Chr15	10,349,289	10,908,248	558,959
Chr16	9,515,642	12,357,011	2,841,369
Chr17	10,763,507	14,039,970	3,276,463
Chr18	12,921,901	16,617,563	3,695,662
Chr19	9,753,755	14,432,088	4,678,333
Chr20	11,800,004	20,656,156	8,856,152
Unanchored	34,820,960		
Total	273,422,085	314,343,867	76,477,398

Supplementary Table 8. Statistical summary of protein-coding genes predicted in *Cucurbita moschata* PKUMo and Rifu genomes.

	Gene Number	mRNA Number	CDS Length (bp)	Exon Length (bp)	Intron Length (bp)	Exons per Gene
PKUMo	28,594	28,594	1362.32	244.66	405.88	5.57
Rifu	32,023	32,023	1235.41	255.45	440.03	5.86

Supplemental Table 9. Genome annotation completeness evaluation of *Cucurbita moschata* PKUMo and Rifu using Benchmarking Universal Single-Copy Orthologs (BUSCO).

	PKUMo		Rifu	
Type	Number	Percentage (%)	Number	Percentage (%)
Complete BUSCOs	1549	96.0	1514	93.80
Single copy BUSCOs	1322	81.9	1288	79.80
Multi copy BUSCOs	227	14.1	226	14.00
Fragmented BUSCOs	22	1.4	53	3.28
Missing BUSCOs	43	2.7	47	2.91
Number of BUSCOs	1614	-	1614	-

Supplementary Table 10. Functional annotation statistics of PKUMo genes annotated in the EggNOG database.

Database	Number	Percentage (%)
EggNOG	27,357	95.06
GOs	15,054	52.31
KEGG_ko	12,993	45.15
KEGG_Pathway	8,191	28.46
KEGG_Module	3,280	11.40
KEGG_Reaction	3,497	12.15
KEGG_rclass	3,313	11.51
BRITE	12,993	45.15
KEGG_TC	1,314	4.57
CAZy	627	2.18
PFAMs	23,998	83.38

Supplementary Table 11. Summary of interspersed repetitive elements predicted in the PKUMo genome.

Class	Number	Length	Percentage (%)
Retroelements	167,532	74,737,886	23.78
SINEs	1,772	916,970	0.29
LINEs	7,765	2,250,672	0.72
LTR/ Copia	51,180	31,076,956	9.89
LTR/ Gypsy	48,327	23,581,853	7.50
DNA transposons	7,936	1,559,635	0.50
hobo-Activator	2,259	460,078	0.15
MULE-MuDR	4,029	697,856	0.22
Unclassified	148,963	51,277,873	16.31
Total		127,575,394	40.58

Supplementary Table 12. Chromosomal rDNA copy number in PKUMo genome**.**

Chr	5S	45S
Chr01	0	263
Chr04	257	0
Chr10	0	442
Chr11	0	533
Chr12	0	774
Chr16	0	108
Chr20	1035	0

Supplementary Table 13. Centromeric regions of each chromosome in the PKUMo genome.

Chr	Start	End	Length (bp)
Chr01	6,747,320	7,193,116	445,796
Chr02	6,885,941	7,366,653	480,712
Chr03	9,058,592	9,696,879	638,287
Chr04	11,438,587	11,914,214	475,627
Chr05	6,856,383	7,309,633	453,250
Chr06	5,433,199	6,005,724	572,525
Chr07	6,041,103	6,194,366	153,263
Chr08	7,332,548	7,656,824	324,276
Chr09	8,882,983	9,310,383	427,400
Chr10	8,417,253	8,925,290	508,037
Chr11	7,961,252	8,570,979	609,727
Chr12	13,424,596	13,987,124	562,528
Chr13	2,862,934	3,304,734	441,800
Chr14	7,337,996	7,890,634	552,638
Chr15	6,637,660	7,124,732	487,072
Chr16	7,328,816	7,481,707	152,891
Chr17	6,367,971	6,751,563	383,592
Chr18	8,043,130	8,544,845	501,715
Chr19	8,474,522	8,951,395	476,873
Chr20	10,463,749	10,972,134	508,385

Supplementary Table 14. Characterization of centromeric satellite monomers in the PKUMo genome.

Monomer	Sequence	NO. copies
**PKUMo**		
**CEN90**	AGAATCATTCAAGCTAGACATGATCAATC<br>TTGCTTGTGGAGTGATTCGAATCTCAAAC<br>AAGTGTTCTTGTCTTGAGATATTCGATCA<br>ACA	5,197
**CEN168**	TAGGGTTTCGATTTGAAGCACTCTTTATC<br>TGTTTTATGCACTCCGTTTCTTCGAAATA<br>CTATTCTAAATCATCAAGCCTAATCATTC<br>CCCACAAAAGTTTCGAAAAACGTCGAAC<br>GTCACTCGGTTTTTTAGCACGTACTTCAA<br>ACGTCCAGTTTAGGGTTTCCGAAA	16,280
**CEN197**	TAATATTGAAGATTTGAAGGTCACGTGTT<br>CGTTCGACCTTCACCACACCTGATTCAA<br>GTAGTATAACTCATAACTTGACTCCTCAC<br>TTTATTTAAAATTAGTGATTATTTCTTTTTA<br>AAATATCAATGAATTAGCTAAATAATTTAC<br>TAATGAGTTTTCATTTTAACGTTAACGGG<br>GATAGCTTAGCTAGGAGGCGG	96
**HZAU***		
**CEN169**	1.TCTGGGACGTTTTTAGTACGTGCCAAA<br>AAAACCGAGTCACGATCGACGTTTTTCG<br>AAAGTTTTGTGAGGAATGGTAAGGCTTG<br>ATGATTTAGAATATTATTTCTAAGAAACG<br>GAGAGCATAAAACTGTAAAAGAGTTCTTC<br>AAATCGAACCCTACTTCGGAAACCCAAA<br>2.ACGTCTATCGTGACTCGGTTTTTTTAGC<br>ACGTACTTCAAACTTCCCAGATTTGGGTT<br>TCCGAAGTAGGGTTCGATTTGAAGAACT<br>CTTTTACTGTTTTATGCTCTCCGTTTCTTA<br>GAAATAATATTCTAAATCATCAAGCCTTA<br>TCATTCCTCAAAAAACTTTCGAAAA<br>3.CCGAGTCACGGTCGACGTTTTTCGAAA<br>GTTTTGTGGAGGAATGATAAGGCTTGAT<br>GATTTAGAATATTATTTCTAAGAAACGGA<br>GTGCATAAAACAGTAAAAGAGTTCTTCAA	126,079

	ATCGAACCCTTCTTCGGAAACCCAAACT<br>GGGAAGTTTGAAGTACGTGCTAAAAAAA<br><br>4.AAGTCTGGGACGTTTGAAGTACGTGTT<br>AAAAAAACCGAGTCACGGTCTTTGTTTTT<br>CGAAAGATTTGTGAGGAATGATTAGGCT<br>TGATGATTTAGAATAGGATTTCGAAGAAA<br>CGGTGTGCATAAAACAGTAAAATAGTTCT<br>TCAAATCGAACCCTACTTCGGAAACCC	
**CEN253**	AAGGTGGGGTGGAGTTGGGTGCCTTCG<br>GGCGTCCAACATGAGCCTCGTCGAGCGT<br>GCGGCGTTGTGGTGACGCCGTCGGGCC<br>ATCCTTGGGGCAGCCCTATGTTTGAAGG<br>CGTGTGGTGCCAGAACATGTTCGGCTGG<br>TTGGAATCGGGTGCCTTTGGGCGTGCGA<br>CATGAGACTAGTTGGGCATGGAGATGTG<br>GCCTTCTAACTTGTGCTGCCTTCGGGAT<br>GGTGGATGGTCGGAACGACGACAAAGC<br>GTGC	10,833
**CEN315**	CCGTTGCCCAGTTTTTTTATAACGTCTCG<br>AATTTCCGTTTGCAAATTAGGGTTTCGAT<br>TTTAGGCACCTTTTTATTGTTTTATACACT<br>CCTTCCCTTCAAAGTTCTATTGCAAACCA<br>TGAGGCTTGACGATTCCCGACAAAATTTT<br>CGTGAACCTCGATCGTTACTCAGTTTTTT<br>TTTAGCACGTGCTTCAAACTTCCCATTTT<br>AGGGTTTCAATTTTAGCACCTTTTTACTG<br>TTTTATACACTCCCTTCCGCCGAAATTCT<br>ATTGCAAATCATGAGGCTTGATCAATCCC<br>CAAACAAATTTTGAAAAACTCCGA	4,789
**CEN324**	ACTGAGTAGCGGTCGGCGTTTTTCGAAA<br>TTCCTGCTCGGAGTGATCGGTAATGATTT<br>AGAATAGAATTTTGAAGAAATGGAGTGCA<br>TAAAACAGTAAAAGAGTGCTTTAAATCGA<br>TTCCCTAACCTGGTTAGTTAGAAGTACGT<br>GCTAAAAAAACTGTGTCACGGTCGACGT<br>TTCTCGAAACTTTTGTTGGGAATGTTTAG<br>TTTAGTCTTGATGATTTAGAATAGAATGT<br>CGAAGAAACGGAGTGTAGAAAAATAGTA<br>GTGCTTAAAATCGAAACCCTAATTTGTGA<br>ACCCTAATCCAGGAAGTTTGAACCACGT<br>GATAAAAAA	4,186

**CEN327**	ACTGAGTAGCGGTCGTCGTTTTTCGAAA<br>TTCCTGTTGGGAGTGATCGGGAAATATG<br>ATTTAGAATAGAATTTGGAAGAAATGGAG<br>TGCATAAAACAGTAAAAGAGTGCTTTAAA<br>TCGAGTCCCTAGCCGGTTAGTTAGAAGT<br>ACGTGCTCAAAAAACCGTGTGACGGTCG<br>ACGTTTCTCGTAACTTTTGTTGGGAATGT<br>TTAGTCTTGTAGATTTGGAATAGAATGTT<br>GAAGAAACGGAGTGTAGAAAAACAGTAA<br>AAGAGTGCATAATATTGAAGCCCTAATTT<br>GTGAACCCTAATCTAGGACGTTTGAACC<br>ACGTGATAAAAAAA	18,634
**CEN654**	ATGAGTAGCGGTCGGCGTTTTTCGAAAT<br>TTCTACACGGAGTGATCGAGAATAATTAT<br>TTAGAATAGAATTTTGAAGAAATTAAGTG<br>TATAAAACAGTAAAATAGTGCTTTAAATC<br>GATTCCCTAACCTGGTTAGTTAGAAATAC<br>GTGCTAAAAAAAGTGTCACGGTCGACGT<br>TTCTCGAAACTTTTGTTGGGAATGTTTAG<br>TTTAGTCTTGATGATTTAGAATAGAATGT<br>CAAAGAAACGGAGTGTAGAAAAATAGTA<br>AAAGAGTGCTTAAAATCGAAGCCCTAATT<br>TGTGAACCCTAATCCAGGACGTTTGAAC<br>CACGTGATAAAAAAACTGAGTAGCGGTC<br>GGCGTTTCTCGAAATTCCTGCTCGGAGT<br>GATCGGGAATAATGATTTAGAATAGAATT<br>TTGAAGAAATGGAGTGCATAAAACAGTAA<br>AAGAGTGCTTTAAATCGATTCCCTAACCT<br>GGTTAGTTAGAAGTACGTGCTAAAAAAAC<br>TGTGTCACGGTCGACGTTTCTCGAAACT<br>TTTGTTGGGAATGTTTAGTTTAGTCTTGA<br>TGATTTAGAATAGAATGTCAAAGAAACGG<br>AGTGTAGAAAAAAAGTAAAAGAGTGCTTA<br>AAATCGAAGCCCTAATTTGTGAACCCTAA<br>TCCAGGACGTTTGAACCACATGA	1,953

***** Data were obtained from Table S16 in the supplementary materials of Zeng, Q. *et al*. (2024).

Supplementary Table 15. General characteristics of full-length long terminal repeat retrotransposons (LTR-RTs) in the PKUMo genome.

Class	Full length LTR-RTs
Total Length (bp)	6,355,200
Total Number	1,192
Min Length (bp)	1,212
Max Length (bp)	17,540
Average Length (bp)	5,331.54
Percentage of Sequence (%)	2.04

Supplementary Table 16. Characterization of different mutant types of butternut squash in M2 families

Characterization of different mutant types of butternut squash in M2 families			
Categories	Phenotypic variations	Number of mutant	Mutation rate(%)
Leaf morphology	wrinkle. Leaf smaller. et al	35	4.375
Leaf color	Yellowing. albino.	26	3.25
Plant architecture	Dwarf. compact. weak. abnormal growing point. etc.	52	6.5
Fertility	Male sterility	2	0.25
Fruit shape	Elongated fruit, shorter fruit	5	0.625
Floral organ morphology	Smaller flower	4	0.5
total		124	15.5

Supplementary Table 17. Single-nucleotide polymorphism (SNP) statistics in M1 and M2 mutants.

	AC	AG	AT	CG	CT	GT	Ts	Tv
M1	19,944	62,202	17,995	13,714	61,894	19,855	124,096	71,508
M2	19,780	58,601	17,526	13,471	58,786	19,521	117,387	70,298

Supplementary Table 18. *mSq* is a recessively inherited mutation

F_2 population	Total No. plants	No. normal plants	No. *mSq* plants	Expected ratio	χ2	P-value
A	112	86	26	3：1	0.1905	0.7547
B	133	100	33	3：1	0.0025	0.9718
C	180	134	46	3：1	0.0296	0.9035
